# Influence of *d*‐Orbital Occupancy on Ligand Conformation and Crystal Packing in a Series of Mononuclear Transition Metal Complexes

**DOI:** 10.1002/open.70304

**Published:** 2026-09-23

**Authors:** Regan F. Soballa, James P. Flood, Weibin Liang, Christopher E. Karlsen, Judith Stuparu, Ross A. Shalliker, Feng Li

**Affiliations:** ^1^ School of Science Western Sydney University Penrith NSW Australia

**Keywords:** crystal engineering, *d‐*orbital occupancy, metallosupramolecular, transition metal complexes, tripodal ligand

## Abstract

A series of mononuclear metal complexes were synthesised incorporating either Mn(II), Co(II), Ni(II), Cu(II), or Zn(II) metal ions in conjunction with a newly reported ligand, tris((((1‐phenyl‐1*H*‐imidazol‐4‐yl)methylene)amino)ethyl)ethane‐1,2‐diamine (**L**). Each of the presently reported metal complexes was characterised in solution and in the solid state. Single‐crystal X‐ray diffraction studies were used to examine how changes in the coordination sphere (and *d*‐orbital occupancy) of each metal ion modify the crystal packing motif, ligand torsion, and the spatial orientation of pendant phenyl groups in the metal complexes. The results presented within demonstrated that the complexation of metal ions bearing different *d*‐orbital occupancies generated separate packing motifs except for the isostructural Co(II) and Zn(II) complexes. These differing motifs gave rise to small variations in the octahedral distortion parameters, ligand arm torsion, and the positions of the pendant phenyl groups across the series. These findings illustrate how structural and electronic differences, arising from the choice of metal ion and the atomic composition of pendant groups, influence ligand conformation and crystal packing within mononuclear systems. Such simple mononuclear complexes may therefore provide a foundation upon which larger, more elaborate metallosupramolecular assemblies can be rationally constructed.

## Graphical abstract

Five new mononuclear complexes of the form [M**L**]X_2_ are reported herein. Structural changes and effects on crystal packing arrangements caused by octahedral distortion in the coordination sphere have been studied.

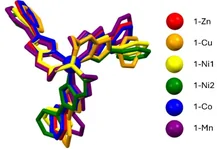

## 1 Introduction

Constructing discrete and structurally well‐defined metallosupramolecular architectures through pre‐programmed combinations of metal ions and ligands has unearthed a rich range of self‐assembled architectures [1, 2, 3, 4, 5, 6, 7, 8]. Metal‐directed self‐assembly strategies relies on key features such as kinetic and thermodynamic control that intrinsically influence the transient and reversible nature of coordination bonds and non‐covalent interactions reflected in the structural and functional aspects of various metallosupramolecular systems [9, 10, 11, 12]. By leveraging small changes in the structural and/or electronic design of metal complexes, e.g., directional bonding preferences and coordination geometries of metal centres, the donor properties and structure of the ligands, etc., the properties and structures of the resulting architectures can be modulated [13, 14, 15, 16]. Alternatively, the atomic composition and spatial arrangement of these simpler components, e.g., coordinating moieties to metal ions, *d*‐orbital occupancy and octahedral distortion in (pseudo)‐octahedral coordination environments, etc., can also influence the structure and multifunctionality of sophisticated metallosupramolecular architectures [17, 18, 19, 20, 21, 22, 23, 24, 25, 26, 27, 28, 29, 30]. An extensive range of shapes, sizes, and properties have been reported over several decades, whereby the structural and electronic features of the metallosupramolecular systems correlate to unique optical, magnetic, or electrochemical properties for applications in guest encapsulation [18, 31, 32, 33, 34], catalysis [35, 36, 37], optoelectronic devices [38, 39, 40], and chemical sensors [41, 42, 43], among others.

Conformational differences arising from the choice of metal ion in metal‐directed design strategies have been investigated in a number of cases. Wang et al. reported multiple Fe(II/III), Co(II/III), and Ni(I/II) dinuclear metal complexes with different properties upon changing the metal ion [44]. The Fe(II/III) and Co(II/III) complexes featured metal–metal single bonds and a redox‐active ligand, while reduced forms of the Co(II/III) and Ni(I/II) complexes exhibited half‐order and no σ‐bonds, respectively, between the metal ions, as well as varying electron density across the ligand. Boronski et al. reported Fe(II), Co(II), Ni(II), Cu(I), and Cu(II) metal complexes with different mono‐β‐diketonato ligand combinations to form dimers [45]. The varying metal ions, ligands, and solvent compositions led to determining potential catalytic applications for the Fe(II) complex in Kumada−Tamao−Corriu coupling reactions and the Cu(I) and Cu(II) complexes for Cu‐catalysed Ullmann cross‐coupling reactions. Kinzel et al. reported various metal complexes with different effects on reducing carbon dioxide [46]. The Fe(II), Co(II), and Ni(II) complexes with the corresponding redox‐innocent ligand were found to reduce carbon dioxide when adopting *3d* ^6–8^ orbital occupancies, while the Mn(II), Cu(I), and Zn(II) complexes with *3d* ^5^ or *3d* ^10^ orbital occupancies were ineffective. Salojarvi et al. reported a metal complex series with Ti(IV), Zr(IV), V(IV), and Ni(II) complexes absorbing very strongly in the near‐infrared region [47]. While the different *d*‐orbital occupancies and coordination numbers with the ligands induce different magnetic and electrochemical properties, the paramagnetic V(IV) and Ni(II) complexes were found to convert near‐infrared photons into free electrons, which is useful in photoelectrochemical applications, while the diamagnetic Ti(IV) and Zr(IV) complexes lacked that ability.

In particular, our research group investigated a tripodal metalloligand to understand how variation of the metal ion influences the assembly of coordination cages. Initial studies focused on synthesising a high‐spin (HS) Fe(II)‐Pd(II) face‐centred cubic coordination cage followed by a spin‐crossover (SCO) Fe(II)‐Ni(II) analogue [48, 49]. More recently, attention has turned to elucidating how different metal ions modulate the ligand torsion and spatial orientation and position of 4‐pyridyl directional bonding axes, and how these structural features propagate into the architecture of the resulting coordination cages [50]. Although numerous studies have demonstrated that metal ion identity can significantly influence the structure and properties of metal complexes, variations to the ligand design—such as substitution of the terminal 4‐pyridyl groups, offer an additional level of control, with substantial alterations to both structural and functional properties.

**SCHEME 1:**
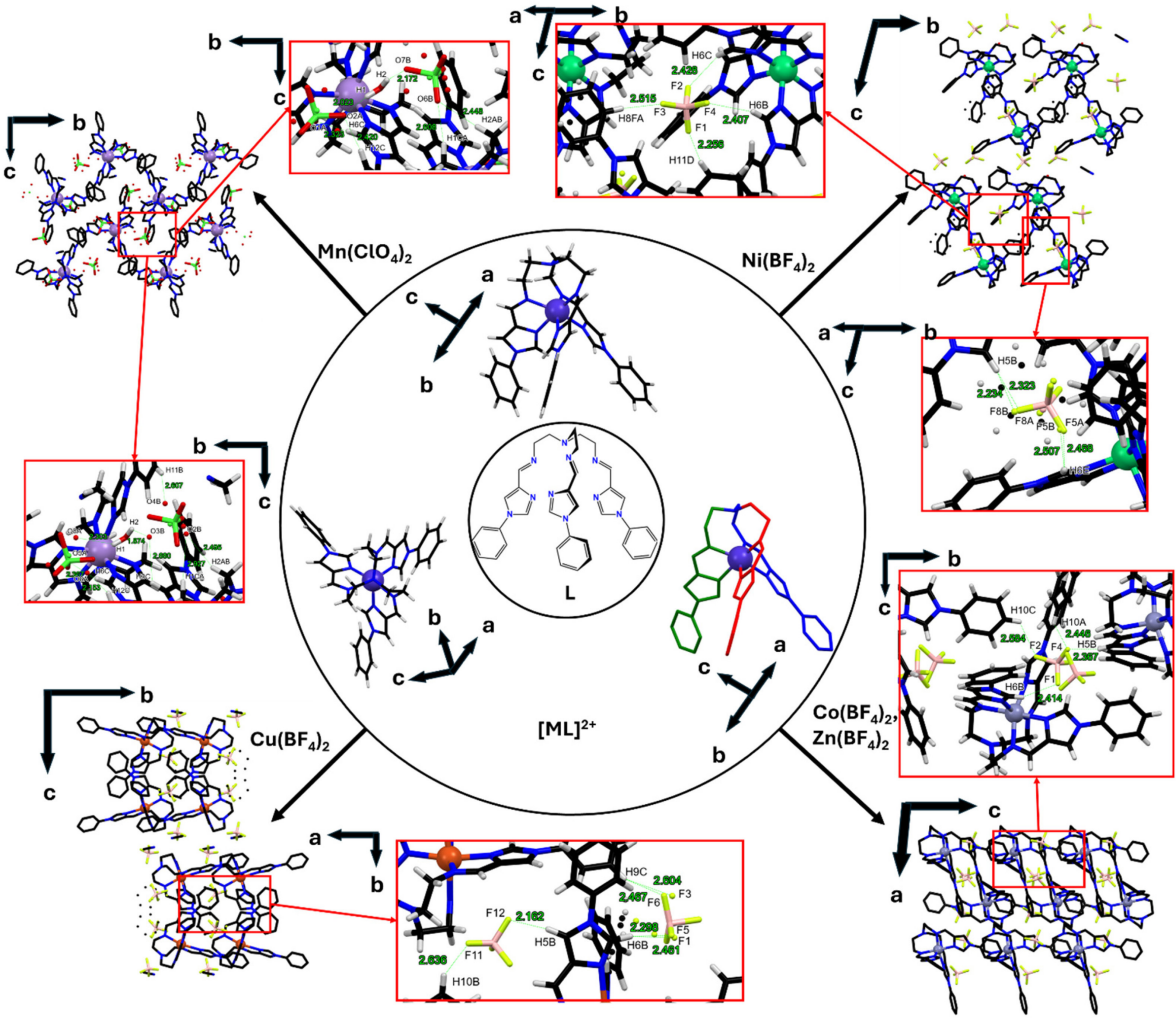
Representation of the metal complex series reported herein. The ligand **L** structure (centre), upon complexation with a metal salt results in a metal complex cation [M**L**]^2+^ with general structures generated from the cif of **1‐Co** from a top (centre, bottom left), and side view (centre, top), and a side view with ligands arms **A** (blue), **B** (green) and **C** (red) (centre, bottom right). Using particular metal salts are found to result in different packing motifs of each metal complex (Mn(ClO_4_)_2_: (top left); Cu(BF_4_)_2_: bottom left; Ni(BF_4_)_2_:top right and; Co(BF_4_)_2_ or Zn(BF_4_)_2_: bottom right). Hydrogen atoms were removed for clarity. Zoomed in representations are also shown for the intermolecular contacts for each packing motif with atom labels (black) and bond distances (green) (Mn(ClO_4_)_2_: top and left; Cu(BF_4_)_2_: bottom; Ni(BF_4_)_2_: top and right; Co(BF_4_)_2_ or Zn(BF_4_)_2_: right). Solvents and anions have been removed in certain instances for clarity. Lone, unbonded atoms are due to phenyl groups or perchlorate anions that are split across different positions, with different hydrogen bonding interactions shown for these split atomic positions.

Herein, we report the synthesis and structure of five mononuclear complexes of the general formula [M^II^ **L**]X_2_ (where M = Mn, Co, Ni, Cu, Zn, X = BF_4_^−^ or ClO_4_^−^, and **L** is tris((((1‐phenyl‐1*H*‐imidazol‐4‐yl)methylene)amino)ethyl)ethane‐1,2‐diamine) (Scheme 1). Single‐crystal X‐ray diffraction analyses were performed to elucidate the influence of metal ion *d*‐orbital occupancy, octahedral distortions, coordination bond lengths, ligand torsion, and the spatial orientation of pendant phenyl groups. Systematic variation in *d*‐orbital occupancy correlates with measurable changes in the positioning and orientation of pendant phenyl groups, which, in turn, may lead to the increased diversity of crystal packing arrangements and intermolecular interaction networks. Notably, these results obtained for the phenyl‐substituted derivatives prove to be markedly different from their 4‐pyridyl analogues, for which the crystal packing motifs are largely conserved, except for the Jahn–Teller distorted Cu(II) complex. The present metal complexes generally displayed reduced octahedral distortion parameters and lower coordination bond lengths relative to the 4‐pyridyl derivatives while maintaining similar periodic trends across the metal series. In contrast, greater variability of the spatial orientation of the phenyl pendant groups and increased ligand torsions are observed in the phenyl‐substituted complexes, albeit with some exceptions. These provide insight into determining substituent effects in other metallosupramolecular systems, particularly in relation to metal‐ion‐dependent structural variations. Further studies will focus on the effects of anions and/or solvent variations, with the aim of probing spin transition behaviours and exploring additional crystal packing motifs in related complexes.

## 2 Results and Discussion

### 2.1 Synthesis and Characterisation of 1‐(phenyl)‐1*H*‐Imidazole‐4‐Carbaldehyde and L

**FIGURE 1:**
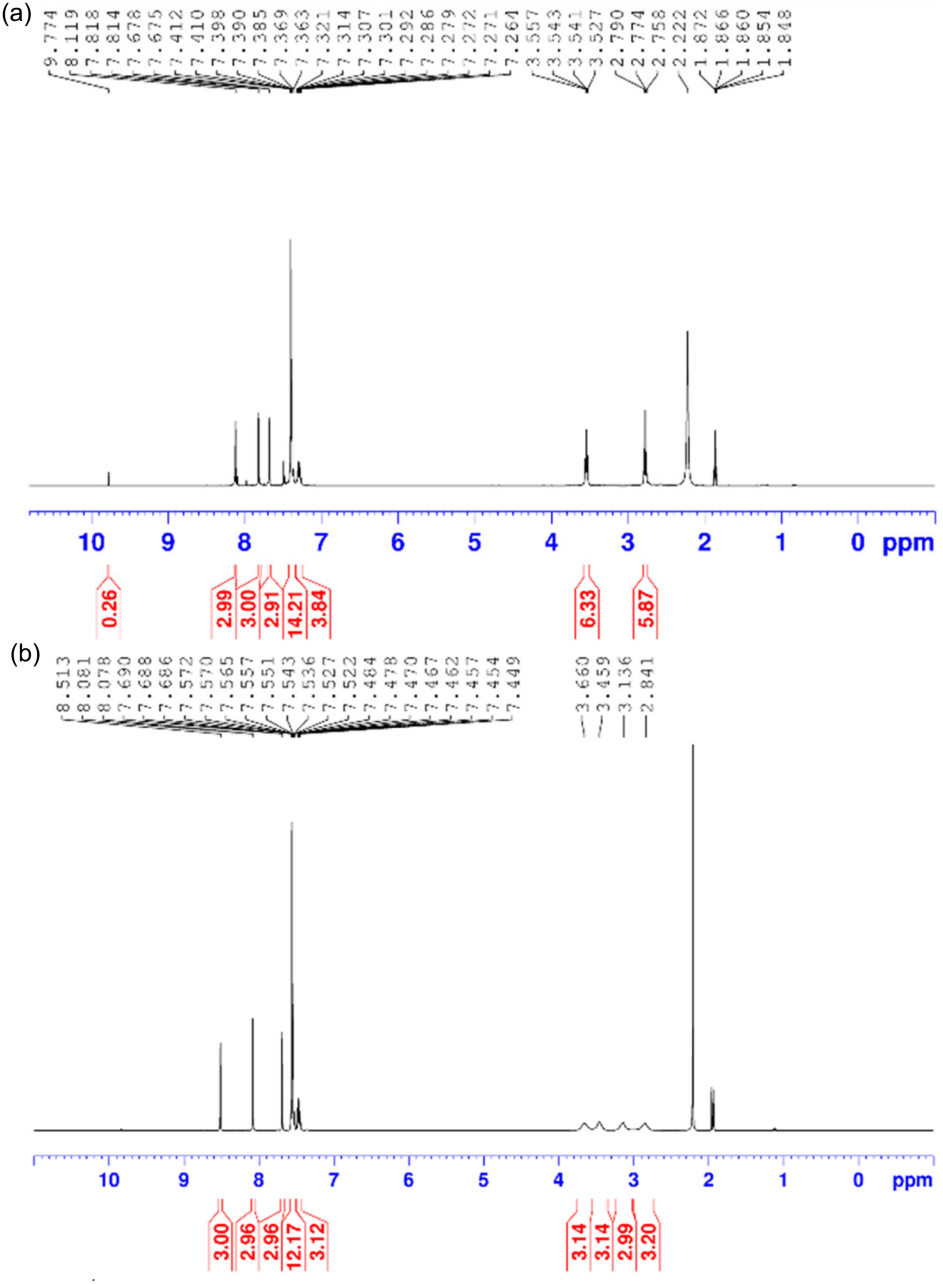
^1^H NMR of (a) crude **L** and (b) **1‐Zn**.

The precursor (1‐(phenyl)‐1*H*‐imidazole‐4‐carbaldehyde) was synthesised by an Ullmann cross‐coupling reaction with iodobenzene and 4‐imidazolecarboxaldehyde. Following isolation and purification of the precursor, a Schiff base condensation reaction was carried out with tris(2‐aminoethyl)amine with an approx. 3:1 ratio in slight excess of precursor, producing **L** as a crude oil, which was used without further purification. Full synthetic procedures can be found in the Supporting Information (pp. S4−5). ESI‐MS, FT‐IR, ^1^H, and ^13^C NMR confirm the successful synthesis of the precursor and **L**. The precursor and **L** were identified by *m/z* ratios of 173.13 and 609.61, corresponding to the [M+H]^+^ ion, respectively (Figures S1, S2). A ^1^H NMR peak at 9.94 ppm and a ^13^C NMR peak at 186.04 ppm correspond to the HC═O proton and carbon atoms, indicating an aldehyde functional group for the precursor (Figures S8, S9). A new ^1^H NMR peak at 8.12 ppm and a ^13^C NMR peak at 156.70 ppm indicate the formation of an imine functional group for **L** (Figure 1a, S10). FT‐IR of the precursor indicated aldehyde C═O and C─H bond vibrations at 1681 and 2746 cm^−1^, respectively (Figure S12). Upon reacting with tris(2‐aminoethylamine), the aldehyde C═O becomes an imine C═N with a new band at 1648 cm^−1^ (Figure S13). A full data analysis can be found in the Supporting Information (pp. S8).

### 2.2 Synthesis and Characterisation of [M^II^L]X_2_ (Where M = Mn, Co, Ni, Cu or Zn and X = BF_4_^−^ or ClO_4_^−^)

All metal complexes were synthesised by the dropwise addition of a dilute solution of the corresponding metal salt to **L** in acetonitrile from which X‐ray quality crystals of each complex were obtained by the slow diffusion of diethyl ether into the acetonitrile solution (pp. S5−7). Each metal complex was then characterised using ESI‐MS, FT‐IR, SEM‐EDS, UV–Vis, STA, PXRD, and SCXRD. **1‐Zn** was further analysed by ^1^H and ^13^C NMR and solid‐state fluorescence studies. The mass spectra obtained for each complex in acetonitrile identified *m/z* values corresponding to both [M**L**]^2+^ and {[M**L**]X}^+^ ions for each complex (Figure S3–S7). The major peaks for **1‐Mn**, **1‐Co**, **1‐Ni**, **1‐Cu,** and **1‐Zn** occurred at a *m/z* ratio of 332.11, 334.11, 333.71, 336.21, and 336.71, respectively, and are in excellent agreement with the expected values for the [M**L**]^2+^ complex ions in each case. FT‐IR indicated bands associated with C─H (3158–2814 cm^−1^), C═N (1644–1638 cm^−1^), and aromatic C═C and C═N (1600–1506 cm^−1^) bond vibrations (Figures S14–‍S18). Backscattering SEM micrographs of the respective crystals for each complex displayed block crystals with the corresponding EDS analysis, indicating the presence of all expected elements, with additional images of each crystal taken with an optical microscope (Figure S19‐S20). (Note: The presence of Si and Al peaks in the EDS arise from the silicon wafer and aluminium stub used to mount the crystals). UV–Visvis spectra obtained for each metal complex indicated a band likely associated with the ligand at approximately 258 nm apart from **1‐Cu** at approximately 253 nm (Figure S21). Further bands can be observed at approximately 467, 569, and 726 nm for **1‐Co**, **1‐Ni,** and **1‐Cu,** respectively, and an additional band at approximately 893 nm for **1‐Ni**, which are likely caused by *d*–*d* electron transitions (Figure ‍2a). STA obtained for **1‐Co**, **1‐Ni**, **1‐Cu,** and **1‐Zn** indicated a series of events associated with residual water loss and decomposition events. (Note: STA was not obtained for **1‐Mn** to avoid the risk of explosion with perchlorate anions). Residual water molecules were observed to be removed at 401 K for **1‐Ni**. An additional endothermic peak appears at 556 K for **1‐Ni**, potentially due to melting or a phase transition, and an additional exothermic peak appears at 502 K for **1‐Cu**, which may be attributed to a phase transition. Melting and decomposition of these samples then occur at around 595–636 K (Figures S22–S25). Experimental PXRD patterns of each sample were compared with the respective simulated powder pattern generated from the unit cell parameters generated from the SCXRD cifs (Figures S26–S30). The spectra obtained indicated good correlation with the SCXRD data for all metal complexes.

**FIGURE 2:**
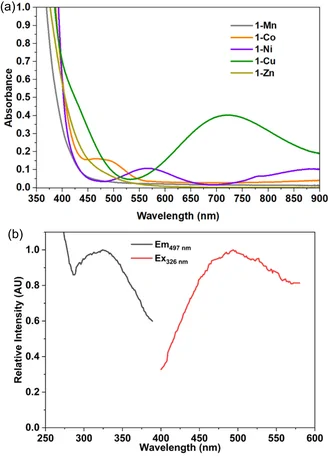
(a) UV–vis spectrum of all metal complexes from 350–900 nm. (b) Solid‐state fluorescence excitation (black) and emission (red) spectra of **1‐Zn**.

### 2.3 NMR and Solid‐State Fluorescence Analysis of 1‐Zn

^1^H NMR of **1‐Zn** identified peaks between 2.84 and 3.66 ppm corresponding to the CH_2_ protons, 7.44 and 8.08 ppm for aromatic protons, and 8.51 ppm for imine protons (Figure 1b). Some peaks for **1‐Zn** were found to have shifted relative to the peaks of **L** (8.12 to 8.51 ppm, 7.82 to 8.08 ppm, and 7.26–7.82 to 7.44–7.57 ppm) (Figure 1a). Peaks for the alkyl CH_2_ protons were found to split as a result of forming the complex (3.54 to 3.66 and 3.44, 2.77 to 3.14 and 2.84 ppm). ^13^C NMR of **1‐Zn** identified peaks of 55.29 and 56.81 ppm corresponding to the CH_2_ protons, between 122.30 and 137.57 ppm for aromatic carbon atoms, and 156.66 ppm for imine carbon atoms (Figure S11). Solid‐state fluorescence studies indicate that upon excitation at approximately 326 nm, crystalline **1‐Zn** displays a fluorescence peak at approximately 497 nm, and the quantum yield was determined to be approximately 10% (Figure 2b). No solution‐state fluorescence was observed for **1‐Zn** in acetonitrile.

## 3 Crystallography

### 3.1 Motifs and Intermolecular Contacts of All [M^II^L]X_2_ Species

Crystal structures obtained at 100 K confirmed that each complex is of the form [M^II^ **L**]X_2_. The respective packing motifs and intermolecular contacts can be found in Scheme 1, while additional crystallographic information can be found in Table S1. Single crystals of **1‐Mn** crystallized in the monoclinic *P2*_1_ */n* space group with one metal complex cation, two perchlorate anions, one acetonitrile molecule and one water molecule. Single crystals of **1‐Ni** crystallized in the triclinic *P1* space group with two complex cations distinguished as **1‐Ni1** and **1‐Ni2**, four tetrafluoroborate anions, one acetonitrile molecule, and one water molecule. Single crystals of **1‐Cu** crystallized in the monoclinic *C2/*c space group with one complex cation, two tetrafluoroborate anions, and one acetonitrile molecule. Single crystals of **1‐Co** and **1‐Zn** crystallized in the monoclinic *P2*_1_ */c* space group with one complex cation and two tetrafluoroborate anions. Each structure has both Δ and Λ optical isomers present in the unit cell, with three imidazole nitrogen and three imine nitrogen donor atoms constructing a distorted octahedral coordination sphere.

Crystal structures of **1‐Co** show complex cations arranged inversely and in an antiparallel manner with hydrogen bonding between all three ligand arms (**A**, **B,** and **C**) and the tetrafluoroborate anions along the crystallographic *b*‐axis (H10B···F4‐B1‐F1···H6B) and the *c*‐axis (H10A···F3‐B1‐F2···H6C). **1‐Zn** crystallizes in a similar way along the *c*‐axis (H10A···F4‐B1‐F1···H6B) and the *b*‐axis (H10C···F2‐B1‐F4···H5B). The crystal packing of **1‐Cu** and **1‐Ni** consists of hydrogen bonding interactions between complex cations, tetrafluoroborate anions, and solvent molecules. The crystal packing of **1‐Ni** consists of rows of complex cations alternating perpendicular to each other along the *c*‐axis with ligand arms **C** and **D** (H6C···F2‐B1‐F1···H11D), the *b*‐axis with ligand arms **B** and **A** (H6B···F4‐B1‐F3···H8FA), and the *a*‐axis with ligand arms **B** and **E** (H5B···F8A‐B2A‐F5A···H6E). (Note: Ligand arms **A**, **B,** and **C** are associated with **1‐Ni1,** while ligand arms **D**, **E,** and **F** are associated with **1‐Ni2**). Additional hydrogen bonds occur due to the splitting of the phenyl group, resulting in similar hydrogen bond distances along the *a*‐axis with ligand arms **B** and **E** (H5B···F8B‐B2B‐F5B···H6E). The major occupancies for the split phenyl group for **1‐Ni1** and **1‐Ni2** are 0.51 and 0.55, respectively, while the minor occupancies are 0.49 and 0.45, respectively. In contrast, the crystal packing of **1‐Cu** consists of complex cations arranged parallel along the *b*‐axis with ligand arms **B** and **C** (H6B···F5‐B1‐F6···H9C), with rows of complex cations arranged perpendicular to each other along the *c*‐axis (H5B···F12‐B2‐F11···H10B). Additional hydrogen bonds occur due to the splitting of the phenyl group, resulting in similar hydrogen bond distances along the *b*‐axis (H6B···F1‐B1‐F3···H9C). The major and minor occupancies for the split phenyl group for **1‐Cu** are 0.51 and 0.49, respectively. In contrast, the crystal packing of **1‐Mn** is different, such that hydrogen bonding is present between all three ligand arms (**A**, **B,** and **C**) of the complex cations, perchlorate anions, acetonitrile, and water molecules. The complex cations are arranged in an antiparallel manner along the *b*‐axis (H6C···O1A‐Cl1A‐O2A···H1‐O1S‐H2···O7B‐Cl2B‐O6B···H2AB) and along the *c*‐axis (O6B···H1CA, H12C···O2A, H11B···O4B‐Cl1B‐O3B···H3C). Additional hydrogen bonds occur due to the splitting of the perchlorate anions, resulting in similar hydrogen bond distances along the *b*‐axis (H6C···O5A‐Cl2A‐O8A···H1‐O1S‐H2···O3B‐Cl1B‐O2B···H2AB) and along the *c*‐axis (O2B···H1CA, H12C···O6A). The perchlorate anion labelled A has occupancies of 0.70 and 0.30, while the perchlorate anion labelled B has occupancies of 0.61 and 0.39.

### 3.2 Structural Comparison of All [M^II^L]X_2_ Species

**FIGURE 3:**
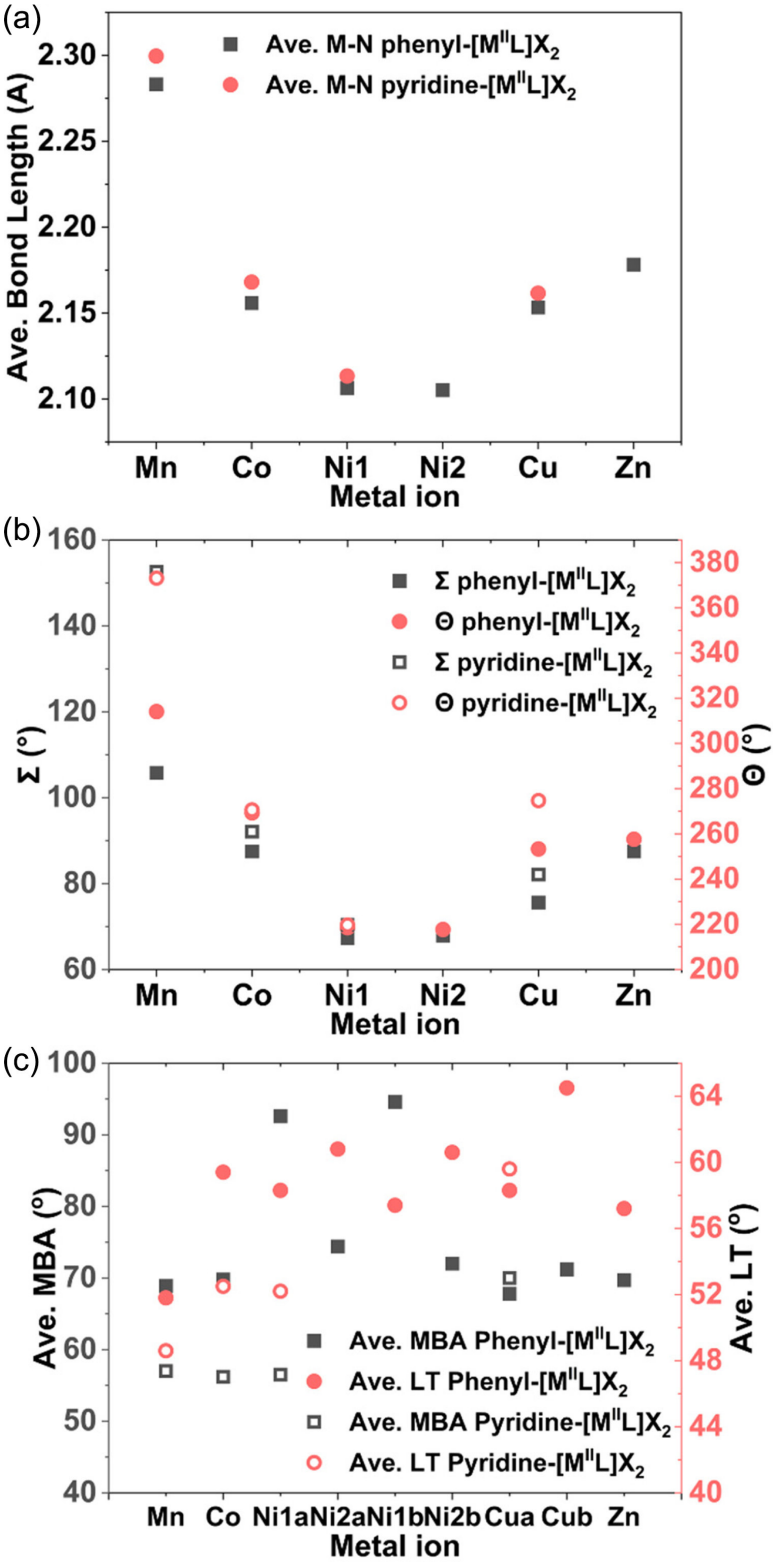
(a) Average metal‐to‐ligand bond distances, (b) Σ and Θ, and (c) MBA and LT, of all phenyl (black) and pyridine (red) metal complexes. The Ni metal centres are distinguished with 1 and 2 to determine which metal centre is being compared in the asymmetric unit respectively, while a and b are used to determine the values involving the major or minor occupancies of the phenyl groups for **1‐Ni1**, **1‐Ni2** and **1‐Cu,** respectively.

The octahedral distortion parameters that measure the average coordination bond distances and the sum of octahedral distortions were calculated for each complex cation using OctaDist (Table 1) [51]. The largest average coordinate bond length is found in **1‐Mn** with 2.28 Å, which is most likely due to a combination of the size of the perchlorate anion, the *d*‐electron configuration of the Mn(II) ion, and the different hydrogen bonding between oxygen or fluorine atoms (Figure 3a). The average coordinate bond length is then observed to decrease to 2.16 Å for **1‐Co** and to 2.11 Å for **1‐Ni1** and **1‐Ni2**. The average coordinate bond length then increases to 2.15 Å for **1‐Cu,** which is likely due to Jahn–Teller distortions. The average coordinate bond length then increases again to 2.18 Å for **1‐Zn**. The angular distortion parameters calculated values of (Σ/Θ) 105.8/314.1°, 87.5/269.3°, 67.3/218.5°, 67.9/217.7°, 75.6/253.3°, and 87.5/257.6° for **1‐Mn**, **1‐Co**, **1‐Ni1**, **1‐Ni2**, **1‐Cu,** and **1‐Zn**, respectively, which follows a similar trend observed for the average coordinate bond lengths obtained for each complex (Figure 3b). Comparison of these values indicates two points: (1) larger paramagnetic metal ions display greater octahedral distortion parameters, with **1‐Mn** demonstrating the largest values and both metal centres in **1‐Ni** demonstrating the lowest values. An exception arises for **1‐Cu** due to Jahn–Teller distortions, and (2) the average coordinate bond length and octahedral distortion parameters of diamagnetic **1‐Zn** increase relative to the Jahn–Teller‐distorted **1‐Cu**, instead demonstrating similar values to **1‐Co**, which may indicate why these two species crystallise in isostructural packing motifs. Similarities occurring for the average coordinate bond length and octahedral distortion parameters obtained for **1‐Co** and **1‐Zn** may indicate potential applications in single‐molecule magnetic behaviours, however; further investigation would be required [6, 30].

**TABLE 1:** Octahedral distortion parameters, ligand torsion, and average mutual bonding axis of [M**L**]X_2_ (**1‐M**) and previously reported structures by our research group for comparison (**2‐M**).

Compound	Average M‐N distances, Å	Σ, °^a^	Θ, °^b^	Average mutual bonding axis, °^c^	Average ligand torsion, °^c^
**1‐Mn**	2.2832(13)	105.8	314.1	68.9	51.8
**1‐Co**	2.1559(15)	87.5	269.3	69.8	59.4
**1‐Ni1** d	2.1064(5)	67.3	218.5	92.6 (94.6)	58.3 (57.4)
**1‐Ni2** d	2.1052(5)	67.9	217.7	74.4 (72.0)	60.8 (60.6)
**1‐Cu**	2.1533(5)	75.6	253.3	67.8 (71.2)	58.3 (64.5)
**1‐Zn**	2.1782(14)	87.5	257.6	69.7	57.2
**2‐Mn** [50]	2.2996(17)	152.6	373.1	57.0	48.6
**2‐Co** [50]	2.1681(17)	92.1	270.6	56.2	52.5
**2‐Ni** [50]	2.1134(18)	70.4	219.7	56.5	52.2
**2‐Cu** [50]	2.1616(15)	82.1	274.7	70.0	59.6

a The parameter Σ is calculated by the sum of the deviations of each ligand‐metal‐ligand angle from the standard 90° octahedron angles.

b The parameter Θ is calculated from the sum of deviations of each ligand‐metal‐ligand angle projected onto the opposing trigonal face of a perfect octahedron from the standard 60° angles.

c Values in brackets for **1‐Ni1**, **1‐Ni2** and **1‐Cu** are due to the splitting of one phenyl arm causing different spatial orientation of pendant phenyl groups and ligand torsion values.

d **1‐Ni1** and **1‐Ni2** represent the two crystallographically distinct metal centres in the asymmetric unit of **1‐Ni**.

To compare how the metal ion size affects the structure of the ligand backbone in these complexes, the structural parameters of mutual bonding axis (MBA) and ligand torsion (LT) have been calculated for the presently reported metal complexes. Further details regarding the calculation of these parameters can be found in our previous works [8, 48, 50] in the supplementary information and as a graphical representation (Figure S31). Previous studies have examined the relationship between these structural features and the bonding directions of secondary coordination domains in a series of similar metal complexes that may act as metalloligands. Elimination of the secondary coordination domains in the present complexes reveals key differences in the MBA and LT between the two series of structures. However, to avoid confusion about the fact that there are no secondary coordination domains in the present complexes, the MBA value will be used as an indication to instead quantify the ‘spread’ of pendant phenyl groups. The MBA and LT calculated for each metal complex (MBA/LT) indicate the values 68.9/51.8°, 69.8/59.4°, 92.6/58.3°, 74.4/60.8°, 67.8/58.3°, and 69.7/57.2° for **1‐Mn**, **1‐Co**, **1‐Ni1**, **1‐Ni2**, **1‐Cu,** and **1‐Zn,** respectively (Figure ‍3c). The second set of MBA and LT values for **1‐Ni1**, **1‐Ni2,** and **1‐Cu,** caused by one phenyl group being split across a second position, are 94.6/57.4°, 72.0/60.6°, and 71.2/64.5°, respectively.

As the metal ion size reduces, the MBA increases from the smallest value for **1‐Mn** to **1‐Ni1** and **1‐Ni2** before decreasing slightly with **1‐Cu**, which is likely a consequence of the Jahn–Teller distortion. MBA values then increase with **1‐Zn**, potentially due to the larger ionic radius of the diamagnetic Zn(II) metal ion. Similar MBA values for **1‐Zn** and **1‐Co** may be reflected in the analogous crystal packing motifs observed for these structures. Additionally, the Ni(II)‐containing complexes seemingly exhibit the largest MBA values for the entire series; however, the values between **1‐Ni1** and **1‐Ni2** are drastically different from one another (Table 1). This is likely due to the positioning and orientation of complex cations, tetrafluoroborate anions, and solvent molecules in the asymmetric unit seemingly leading to the stabilization of two crystallographically distinct conformations. The two MBA values occurring between the main residue and split phenyl rings for **1‐Ni1** and **1‐Ni2** are reasonably similar, indicating that these values aren’t largely affected by the crystallographically distinct environments of these subunits. However, the two MBA values for **1‐Cu** have a larger difference between them, which is most likely due to the Jahn–Teller elongated axis.

Interestingly, the LT trends are different when comparing the two metal centres **1‐Ni1** and **1‐Ni2**, which have similar LT values. When comparing with **1‐Ni1**, the LT increases from **1‐Mn** to **1‐Co** but then slightly decreases to **1‐Ni1**. As a result, **1‐Ni1** has a similar LT value to **1‐Cu**, which then decreases to **1‐Zn**. When comparing with **1‐Ni2**, the LT increases from **1‐Mn** to **1‐Ni2** and then decreasing to **1‐Zn**. However, the LT and MBA trends are similar as **1‐Mn** and **1‐Ni2** have the lowest and highest LT values, respectively. Additionally, the splitting of the phenyl group results in two similar LT values for **1‐Ni1** and **1‐Ni2,** while the two LT values for **1‐Cu** have a larger difference, also likely due to Jahn–Teller distortion. The LT values for **1‐Co** and **1‐Zn** vary slightly, which is likely due to the *d*‐orbital occupancy. Overall, as the metal‐ion size is reduced, the ligand arms tend to spread and twist more, which can greatly affect the crystal packing.

These values are significantly different from that of the nearly analogous and previously reported metalloligand series [50]. Comparison of these values occurring for the pyridine‐containing metal complexes demonstrates a relatively larger set of octahedral distortion parameters and slightly larger metal–ligand bond lengths. However, these values still follow the same trend as the presently reported metal complex series. Despite this, the pyridine complexes display a set of lower MBA values compared to the phenyl‐containing derivative, except for **2‐Cu,** which is larger compared to **1‐Cu**, as well as the much smaller increase from **2‐Co** to **2‐Ni**, when compared to similar measurements between **1‐Co** and **1‐Ni1**/**1‐Ni2**. Additionally, the LT angles are generally lower for the pyridine derivatives, again with the exception of **2‐Cu** and the small decrease from **2‐Co** to **2‐Ni**. This may be due to the replacement of a 4‐pyridyl nitrogen with an aromatic C─H moiety thereby reducing the hydrogen bond acceptor ability of terminal subunits.

Furthermore, the pyridine derivatives, **2‐Mn**, **2‐Co,** and **2‐Ni**, were found to all crystallize in the monoclinic *P2*_1_ */c* space group featuring hydrogen bonding and different packing arrangements involving the 4‐pyridyl nitrogen, except for **2‐Cu** crystallizing in a triclinic *P1* space group, possibly due to the Jahn–Teller elongated axis. By simply removing the 4‐pyridyl nitrogen, which may act as a hydrogen bond acceptor, the LT and MBA values have been affected in the phenyl‐substituted analogues, resulting in an array of different crystal packing motifs for **1‐Mn**, **1‐Ni,** and **1‐Cu** with a separate isostructural packing arrangement for **1‐Co** and **1‐Zn**. Replacing the hydrogen bond acceptor ability of the 4‐pyridyl moiety with the hydrogen bond donor ability of a C─H moiety, different connectivity between neighbouring complexes has been achieved, potentially contributing to different packing‐reliant functionality.

## 4 Conclusion

In summary, we present the formation of a series of metal complex species of the form [M^II^ **L**]X_2_ (where M = Mn, Co, Ni, Cu, and Zn, X = BF_4_^−^ or ClO_4_^−^, and **L** = tris((((1‐phenyl‐1*H*‐imidazole‐4‐yl)methylene)amino)ethyl)ethane‐1,2‐diamine). The complexation with increasingly larger transition metals resulted in changes to the octahedral distortion parameters, spatial orientation of pendant phenyl groups, and ligand torsion. [Mn**L**](ClO_4_)_2_ was found to bear the smaller ‘spread’ of pendant phenyl groups and the lowest ligand torsion, while [Ni**L**](BF_4_)_2_ was found to bear the largest of these values between the two crystallographically‐independent metal centres resolved for this structure. **1‐Mn**, **1‐Ni,** and **1‐Cu** were found to crystallize into different packing arrangements, except for **1‐Co** and **1‐Zn,** which displayed isostructural packing motifs. These results illustrate how crystal packing effects and the subtle modulation of intermolecular interconnectivity within the crystal phase may play a critical role in the metal‐directed self‐assembly of non‐covalently interlocked structures through their influence on intermolecular interactions, solubility, and other chemical characteristics of the complex.

## Author Contributions

**Regan F. Soballa**: methodology, formal analysis, investigation, writing – original draft, review and editing, visualisation. **James P. Flood**: formal analysis, investigation, writing – review and editing, visualisation. **Weibin Liang**: investigation, writing – review and editing. **Christopher E. Karlsen**: investigation. **Judith Stuparu**: investigation, writing – review and editing. **Ross A. Shalliker**: writing – review and editing, supervision. **Feng Li**: conceptualisation, writing – review and editing, visualisation, supervision, project administration.

## Funding

The authors have nothing to report.

## Conflicts of Interest

The authors declare no conflicts of interest.

## Supporting information

Experimental procedures, ESI‐MS, NMR, FT‐IR, SEM‐EDS, Optical Microscopy, UV‐Vis, STA, PXRD and additional crystallographic information.

## Data Availability

The data that support the findings of this study are available within the article and its Supporting Information. Additional data are available from the corresponding author upon reasonable request. The crystallographic data for **1‐Mn**, **1‐Co**, **1‐Ni**, **1‐Cu**, and **1‐Zn** have been deposited with the Cambridge Crystallographic Data Centre (CCDC) under deposition numbers 2565196–2565200, respectively. These data can be obtained free of charge from the joint CCDC/Fachinformationszentrum Karlsruhe (FIZ Karlsruhe) Access Structures service.
