# Supplementary material for: Influence of d‐Orbital Occupancy on Ligand Conformation and Crystal Packing in a Series of Mononuclear Transition Metal Complexes
Source: ChemistryOpen. 2026 Sep 23;15(10):e70304. doi: 10.1002/open.70304 (PMC13601747; doi:10.1002/open.70304)
Supplement: Supplementary file 1 [file OPEN-15-e70304-s001.pdf]

Influence of *d*-Orbital Occupancy on Ligand Conformation and Crystal Packing in a Series of Mononuclear Transition Metal Complexes

Regan F. Soballa,[a] *James P. Flood,*[a] *Weibin Liang,*[a] *Christopher E. Karlsen,*[a] *Judith Stuparu,*[a] *Ross A. Shalliker* [a] *and Feng Li* [a]*

[a] Western Sydney University, School of Science, Penrith, NSW, 2751, Australia

Corresponding author, feng.li@westernsydney.edu.au.

Table of Contents

Experimental 3

Results and Discussion for 1-(phenyl)-1*H*-imidazole-4-carbaldehyde and L 8

ESI-MS 9

NMR 13

FT-IR 15

SEM-EDS 19

Optical Microscopy 20

UV-Vis 21

STA 22

PXRD 24

Crystallographic Information 27

References 29

Experimental

General Techniques

All reagents were obtained from commercial suppliers and used without further purification. Electrospray-ionisation mass spectrometry (ESI-MS) was performed on a Waters Xevo TQ-XS Mass Spectrometer operating in positive ion mode with samples dissolved in CH_3CN. Nuclear magnetic resonance (NMR) spectra were obtained using a Bruker Advance 400 MHz magnet operating at room temperature with samples dissolved in either $CDCl_3$ or CD_3CN. Scanning electron microscopy (SEM) and energy dispersive X-ray spectroscopy (EDS) spot analyses were obtained using a JEOL 6510LV instrument operating with a chamber pressure of 40 or 44 Pa and an accelerating voltage of 25 kV. Samples were carefully mounted on a silicon wafer attached to an aluminium stub with double-sided conductive carbon tape. These samples were then imaged uncoated. EDS spot analysis was obtained using the Moran Scientific microanalysis software package with a silicone drift detector. Fourier Transform-Infrared (FT-IR) measurements were obtained on a Bruker Vertex 70 with diamond crystal ATR. SCXRD measurements for each metal complex were obtained from either the MX1 beamline at the Australian Synchrotron using silicon double crystal monochromated radiation[1] (λ = 0.71073 Å) or from an Oxford Gemini S Ultra instrument outfitted with a MoKα radiation source (λ = 0.71073 Å). At the beamline, the XDS software was used for data integration, processing and scaling. The empirical absorption correction was then applied at the synchrotron using SADABS. The structures were then solved by ShelXT using the intrinsic phasing method. The full-matrix least-squares refinement was then used with various SHELX programs via the Olex2 interface.[2-5] All atoms were refined anisotropically and hydrogen atoms were included in idealized positions and refined using a riding model. Simultaneous thermal analysis (STA) was either obtained from a Netzsch STA-449F3 Jupiter or a Netzsch STA 449C Jupiter instrument for all metal complex species except those containing perchlorate anions to avoid any risk of explosion. A small quantity of sample was gently placed into a pierced aluminium crucible. These samples were then scanned between 300-850 K at a scan rate of 10 K min^{-1} under a continuous nitrogen gas flow at 25 mL min^{-1}. UV-Vis spectra were obtained on a Varian Cary 100 spectrophotometer between 900-200 nm at a scan rate of 600 nm min^{-1}. All samples were dissolved in CH_3CN and added directly into 1 cm quartz cells. Powder X-ray diffraction (PXRD) data was obtained on a Bruker D8 Advance Powder Diffractometer using either a CoKα source (λ = 1.7890 Å) or a CuKα source (λ = 1.5406 Å). Samples were either mounted on a silicon low background XRD plate and measured across the 2Θ range of 5−60° with a step size of 0.02° with a step time of 1.5 s or mounted onto a silicon low background XRD plate with two Kapton foil sheets and measured across the 2Θ range of 5−60°

with a step size of 2.5° with a step time of 2.5 s. Solid-state fluorescence studies were conducted on an Edinburgh instruments FS5 spectrofluorometer. A Xe lamp was used to excite crystalline samples that were placed in a sample holder with a quartz plate and in a perpendicular position. All excitation measurements were each conducted with excitation and emission bandwidths of 1 nm. All emission measurements were each conducted with excitation and emission bandwidths of 1 nm and 0.1 or 1 nm respectively.

Caution: Perchlorate salts are explosive and should be handled with care in a fume hood fitted with a blast shield.

Synthesis of 1-(phenyl)-1*H*-imidazole-4-carbaldehyde:

The precursor was obtained using a modified previously reported procedure.[6] 4-imidazolecarboxaldehyde (2.58 g, 26.9 mmol), copper(I) iodide (0.68 g, 3.55 mmol) and potassium carbonate (36.38 g, 0.263 mol) were added to DMSO (100 mL). The reaction mixture was then evacuated under vacuum and flushed with nitrogen gas 3 times before being heated to 130°C. Over the following two days, a solution of iodobenzene (16.02 g, 78.5 mmol) in DMSO (200 mL) was slowly added to the reaction mixture. Following this, the mixture was then allowed to cool, before being passed through a vacuum followed by a celite filter under vacuum. The remaining solids removed in the filtration process were washed with ethyl acetate (200 mL). Disodium ethylenediaminetetraacetic acid (Na_2H_2EDTA, 1.69 g, 5.78 mmol) and water (200 mL) were then added to the filtrate, and the mixture was then extracted into ethyl acetate (500 mL). The combined organic phases were then washed with water (500 mL), followed by brine (100 mL), dried and evaporated under reduced pressure to yield an off-white crystalline powder. The crystalline powder was recrystallized from ethanol and then slow evaporation from acetone to yield the product as white crystals. Yield = 1.46 g, 32.5%. ^{1}H NMR ($CDCl_3$, 400 MHz) δ (ppm); 9.94 (d, 1H), 8.02 (s, 1H), 7.97 (s, 1H), 7.53-7.55 (m, 2H), 7.45-7.47 (m, 3H); ^{13}C NMR ($CDCl_3$, 100 MHz) δ (ppm); 186.04, 142.94, 137.26, 136.13, 130.17, 128.70, 123.54, 121.69. ESI-MS (positive ion mode, CH_3CN, *m/z*) calcd. for $[M+H]^+$, 173.07, found; 173.13. v_{max}/cm^{-1}: 3119, 3052, 3021, 2923, 2854 (Ar. C-H), 2813, 2746 (Aldehyde C-H), 1681 (Aldehyde C=O), 1600, 1538, 1502 (Ar. C=N and C=C)

Synthesis of tris((((1-phenyl-1*H*-imidazol-4-yl)methylene)amino)ethyl)ethane-1,2-diamine (L):

Excess of the previously synthesised 1-(phenyl)-1*H*-imidazole-4-carbaldehyde (0.25 g, 1.44 mmol) was dissolved in CH_3CN (10 mL) to which tris(2-aminoethyl)amine (63.5 µL, 0.422 mmol) in CH_3CN (15 mL) was added directly. The solution was then heated to reflux overnight yielding a pale-yellow solution. The resulting solution was then evaporated under reduced pressure to obtain **L** as an orange oil for further reactions. Yield = 0.33 g. ^{1}H NMR (CD_3CN, 400 MHz) δ (ppm); 8.12 (s, 3H), 7.82 (d, 3H), 7.68 (d, 3H), 7.36-7.41 (m, 12H), 7.26-7.32 (m, 3H), 3.54 (t, 6H), 2.77 (t, 6H); ^{13}C NMR (CD_3CN, 100 MHz) δ (ppm); 156.70, 142.24, 136.92, 130.61, 128.34, 121.93, 119.21, 118.11, 60.53, 56.18. ESI-MS (positive ion mode, CH_3CN, *m/z*) calcd. for $[M+H]^+$, 609.32, found 609.61 v_{max}/cm^{-1}: 3106, 3071 (Ar. and imine C-H), 2923, 2852 (alkyl C-H), 1648 (imine C=N), 1598, 1502 (Ar. C=N and C=C)

Note: The yield obtained for **L** above is a crude product. Despite this, the purity was high enough to perform the intended metal complexation without further purification.

Synthesis of tris((((1-phenyl-1*H*-imidazol-4-yl)methylene)amino)ethyl)ethane-1,2-diamine manganese(II) perchlorate (1-Mn):

Tris(2-aminoethyl)amine (13.0 µL, 0.088 mmol) in CH_3CN (5 mL) was directly added to a stirring solution of 1-(phenyl)-1*H*-imidazole-4-carbaldehyde (0.056 g, 0.33 mmol) in CH_3CN (5 mL) and the resulting mixture was then stirred at reflux for 18 hours. Manganese(II) perchlorate hydrate (0.025 g, 0.092 mmol) in CH_3CN (10 mL) was then added dropwise and the solution was allowed to react at room temperature for 3 hours under a N_2 atmosphere. The resulting solution was then passed through a filter and diffused slowly against diethyl ether to obtain **1-Mn** as yellow block crystals. Yield: 0.020 mg, 25.8%. v_{max}/cm^{-1}: 3627, 3544, 3108, 3069, 2965, 2916, 2850 (C-H), 1642 (imine C=N), 1600, 1508 (Ar. C=C, C=N). ESI-MS (positive ion mode, CH_3CN, *m/z*) calcd. for $[ML]^{2+}$, 332.13; found, 332.11.

Synthesis of tris((((1-phenyl-1*H*-imidazol-4-yl)methylene)amino)ethyl)ethane-1,2-diamine cobalt(II) tetrafluoroborate (1-Co):

Tris(2-aminoethyl)amine (12.3 μL, 0.083 mmol) in CH_3CN (10 mL) was directly added to a stirring solution of 1-(phenyl)-1*H*-imidazole-4-carbaldehyde (0.052 g, 0.30 mmol) in CH_3CN (5 mL) and the resulting mixture was then stirred at reflux for 24 hours. Cobalt(II) tetrafluoroborate hydrate (0.023 g, 0.090 mmol) in CH_3CN (10 mL) was then added dropwise and the solution was allowed to heat at reflux for 3 hours under a N_2 atmosphere. The resulting solution was then passed through a filter and diffused slowly against diethyl ether to obtain **1-Co** as orange block crystals. Yield: 0.023 mg, 32.4%. v_{max}/cm^{-1}: 3619, 3121, 2958, 2920, 2856 (C-H), 1640 (imine C=N), 1598, 1508 (Ar. C=C, C=N). ESI-MS (positive ion mode, CH_3CN, *m/z*) calcd. for $[ML]^{2+}$, 334.13; found, 334.11.

Synthesis of tris((((1-phenyl-1*H*-imidazol-4-yl)methylene)amino)ethyl)ethane-1,2-diamine nickel(II) tetrafluoroborate (1-Ni):

Tris(2-aminoethyl)amine (13.4 μL, 0.090 mmol) in CH_3CN (10 mL) was directly added to a stirring solution of 1-(phenyl)-1*H*-imidazole-4-carbaldehyde (0.054 g, 0.31 mmol) in CH_3CN (5 mL) and the resulting mixture was then stirred at reflux for 24 hours. Nickel(II) tetrafluoroborate hexahydrate (0.031 g, 0.092 mmol) in CH_3CN (10 mL) was then added dropwise and the solution was allowed to heat at reflux for 3 hours under a N_2 atmosphere. The resulting solution was then passed through a filter and diffused slowly against diethyl ether to obtaining **1-Ni** as purple block crystals. Yield: 0.027 mg, 35.0%. v_{max}/cm^{-1}: 3567, 3158, 3135, 2925, 2850, 2816 (C-H), 1638 (imine C=N), 1600, 1515 (Ar. C=C, C=N). ESI-MS (positive ion mode, CH_3CN, *m/z*) calcd. for $[ML]^{2+}$, 333.63; found, 333.71.

Synthesis of tris((((1-phenyl-1*H*-imidazol-4-yl)methylene)amino)ethyl)ethane-1,2-diamine copper(II) tetrafluoroborate (1-Cu):

Tris(2-aminoethyl)amine (12.9 μL, 0.087 mmol) in CH_3CN (10 mL) was directly added to a stirring solution of 1-(phenyl)-1*H*-imidazole-4-carbaldehyde (0.054 g, 0.31 mmol) in CH_3CN (5 mL) and the resulting mixture was then stirred at reflux for 3 hours. Copper(II) tetrafluoroborate hydrate (0.023 g, 0.10 mmol) in CH_3CN (10 mL) was then added dropwise and the solution was allowed to heat at reflux for 3 hours under a N_2 atmosphere. The resulting solution was then passed through a filter and diffused slowly against diethyl ether to obtain **1-Cu** as green block crystals. Yield: 0.030 mg, 40.4%. v_{max}/cm^{-1}: 3627, 3140, 2929, 2815 (C-H), 1640 (imine C=N), 1600, 1506 (Ar. C=C, C=N). ESI-MS (positive ion mode, CH_3CN, *m/z*) calcd. for $[ML]^{2+}$, 336.13; found, 336.21.

Synthesis of tris((((1-phenyl-1*H*-imidazol-4-yl)methylene)amino)ethyl)ethane-1,2-diamine zinc(II) tetrafluoroborate (1-Zn):

Tris(2-aminoethyl)amine (12.3 µL, 0.083 mmol) in CH_3CN (5 mL) was directly added to a stirring solution 1-(phenyl)-1*H*-imidazole-4-carbaldehyde (0.057 g, 0.33 mmol) in CH_3CN (5 mL) and the resulting mixture was then stirred at reflux for 18 hours. Zinc(II) tetrafluoroborate hydrate (0.023 g, 0.091 mmol) in CH_3CN (10 mL) was then added dropwise and the solution was allowed to heat at reflux for 3 hours under a N_2 atmosphere. The resulting solution was then passed through a filter and diffused slowly against diethyl ether to obtain **1-Zn** as colourless block crystals. Yield: 0.021 mg, 30.3%. ν_{max}/cm^{-1}: 3616, 3108, 2919, 2854 (C-H), 1644 (imine C=N), 1600, 1508 (Ar. C=C and C=N). ESI-MS (positive ion mode, CH_3CN, *m/z*) calcd. for $[ML]^{2+}$, 336.62; found. 336.71, ^1H NMR (CD_3CN, 400 MHz) δ (ppm); 8.51 (s, 3H), 8.08 (d, 3H), 7.69 (d, 3H), 7.44-7.57 (m, 15H), 3.66 (t, 3H), 3.44 (t, 3H), 3.14 (t, 3H), 2.84 (t, 3H); ^{13}C NMR (CD_3CN, 100 MHz) δ (ppm); 156.66, 137.57, 136.60, 136.46, 130.59, 129.29, 123.25, 122.30, 56.81, 55.29.

Results and Discussion for 1-(phenyl)-1*H*-imidazole-4-carbaldehyde and **L**

ESI-MS, FT-IR, ^{1}H and ^{13}C NMR confirm the successful synthesis of 1-(phenyl)-1*H*-imidazole-4-carbaldehyde and **L**. 1-(phenyl)-1*H*-imidazole-4-carbaldehyde was further analysed by SCXRD. ESI-MS of 1-(phenyl)-1*H*-imidazole-4-carbaldehyde identified a *m/z* ratio of 173.13 corresponding to the $[M+H]^+$ ion, which correlates well with the predicted *m/z* ratio of 173.07 (Figure S1). ^{1}H NMR identifies a peak at 9.94 ppm attributed to the HC=O proton (Figure S8). Peaks between 7.45 and 8.02 ppm are indicative of aromatic protons in the phenyl and imidazole groups. ^{13}C NMR identified a peak at 186.04 ppm attributed to the HC=O carbon atom followed by peaks between 121.69 and 142.94 ppm indicating the phenyl and imidazole carbon atoms (Figure S9). Other peaks are due to $CDCl_3$ at 7.37 and 77.27 in the ^{1}H and ^{13}C NMR spectra, respectively. FT-IR indicated aldehyde C=O and C-H bond vibrations due to a strong narrow band at 1681 cm^{-1} and a moderate narrow band at 2746 cm^{-1} respectively (Figure S12). The peak at 3354 cm^{-1} is most likely due to O-H bonds vibrations from residual water molecules. Aromatic C=C, C=N and C-H bond vibrations are supported by peaks at 1502, 1538, 1600, 3021, 3052 and 3119 cm^{-1} respectively.

ESI-MS of **L** identified a *m/z* ratio of 609.61 corresponding to the $[M+H]^+$ ion which correlates well with the predicted *m/z* ratio of 609.32 (Figure S2). ^{1}H NMR identifies a peak at 8.12 ppm attributed to the imine CH proton (Figure 1a). Peaks between 7.26 and 7.82 ppm are indicative of aromatic protons in the phenyl and imidazole groups. New peaks at 3.54 and 2.77 ppm are indicative of alkyl C-H protons in an AB system. ^{13}C NMR identified peaks between 156.70 and 118.11 ppm attributed to the phenyl, imidazole and imine carbon atoms as well as peaks at 60.53 and 56.18 ppm attributed to the alkyl carbon atoms (Figure S10). In the ^{1}H NMR spectrum, a peak at 9.77 ppm is likely due to residual 1-(phenyl)-1*H*-imidazole-4-carbaldehyde as excess 1-(phenyl)-1*H*-imidazole-4-carbaldehyde was present to ensure the formation of the tri-substituted ligand but results in the product as a crude oil. This is supported by ^{13}C NMR which identified a peak at 186.31 ppm that corresponds to the HC=O of residual 1-(phenyl)-1*H*-imidazole-4-carbaldehyde. As **L** couldn't be isolated, it makes sense that the sample is impure with residual 1-(phenyl)-1*H*-imidazole-4-carbaldehyde. FT-IR indicated bands at 3106 and 3071 cm^{-1} caused by imine and aromatic C-H bond vibrations respectively along with bands at 2923 and 2852 cm^{-1} from alkyl C-H bond vibrations and bands at 1598 and 1502 cm^{-1} corresponding to aromatic C=C and C=N bond vibrations (Figure S13). The removal of C=O bond vibrations and the presence of a new band at 1648 cm^{-1} indicates the presence of a successful Schiff base reaction.

ESI-MS

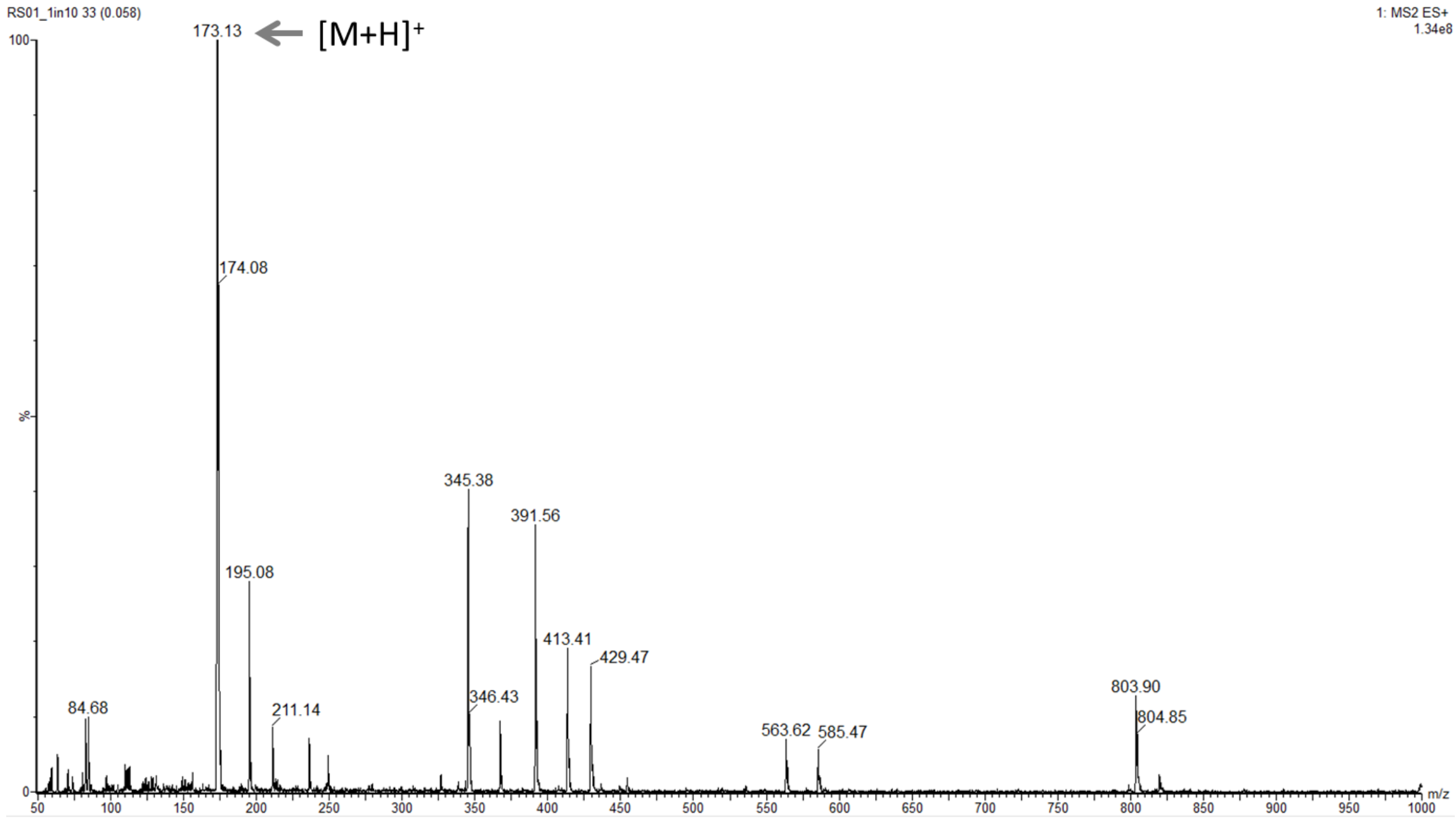

Figure S1: ESI-MS of 1-(phenyl)-1*H*-imidazole-4-carbaldehyde in CH_3CN.

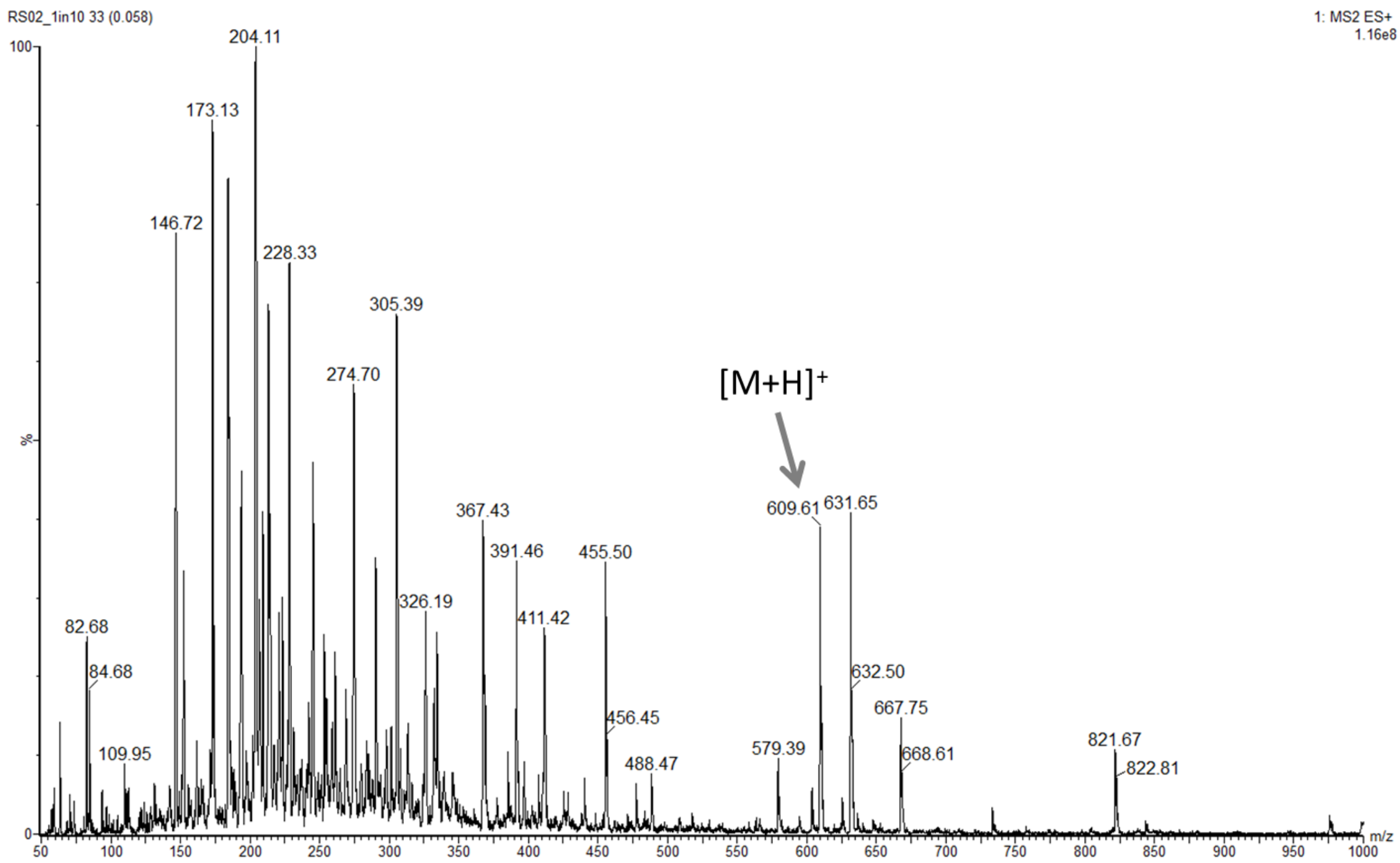

Figure S2: ESI-MS of **L** in CH_3CN.

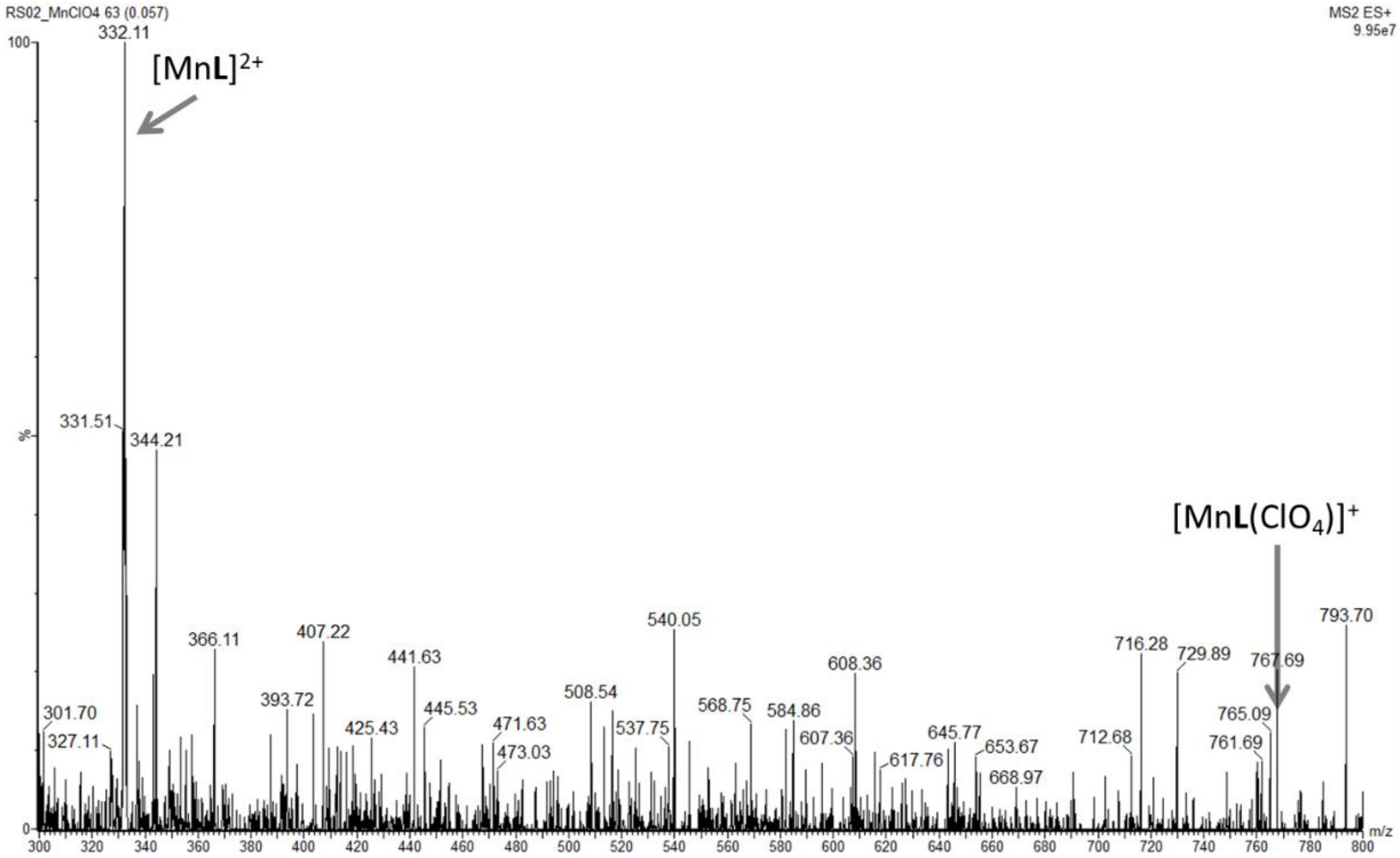

Figure S3: ESI-MS of **1-Mn** in CH_3CN.

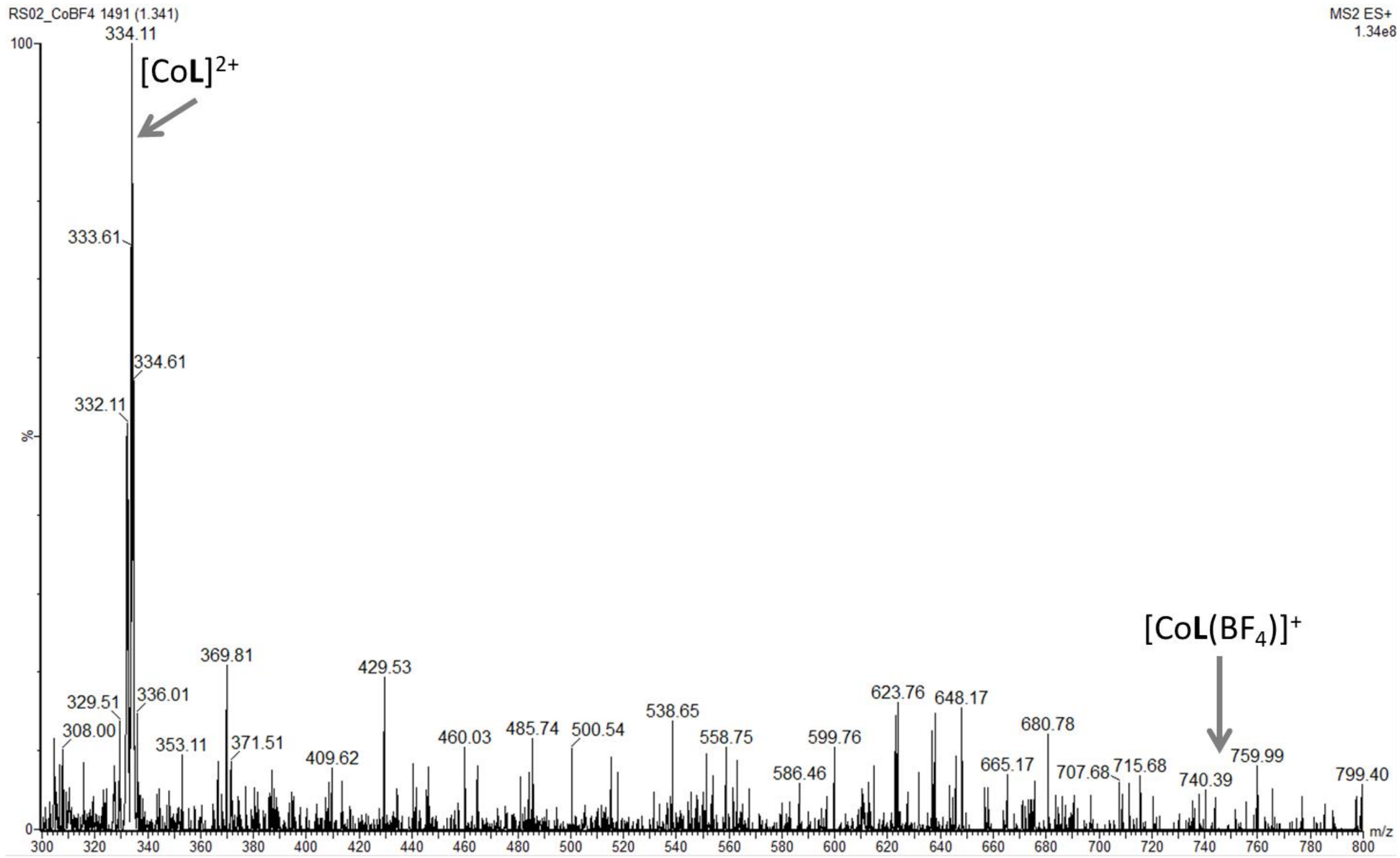

Figure S4: ESI-MS of **1-Co** in CH_3CN.

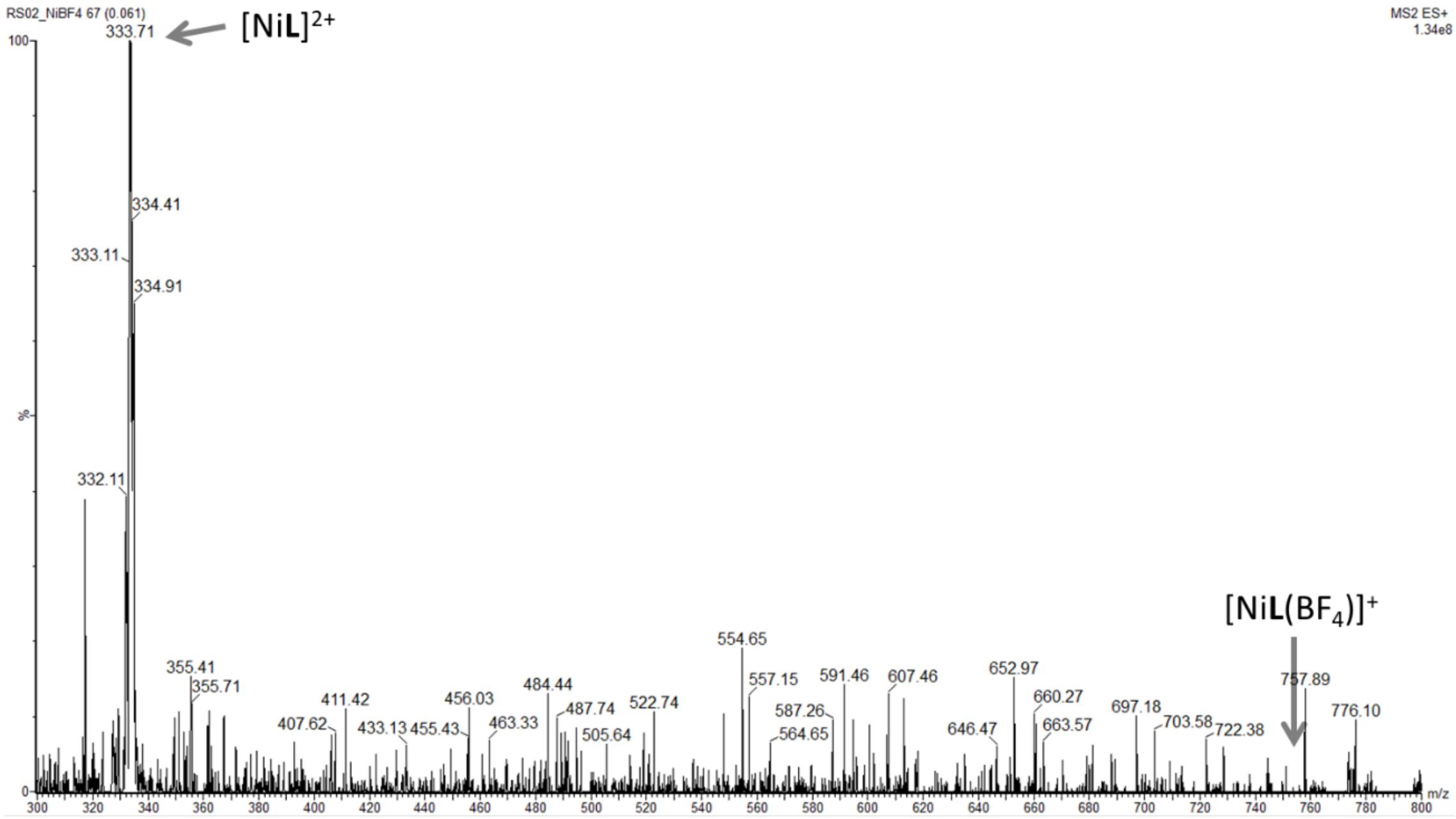

Figure S5: ESI-MS of **1-Ni** in CH_3CN.

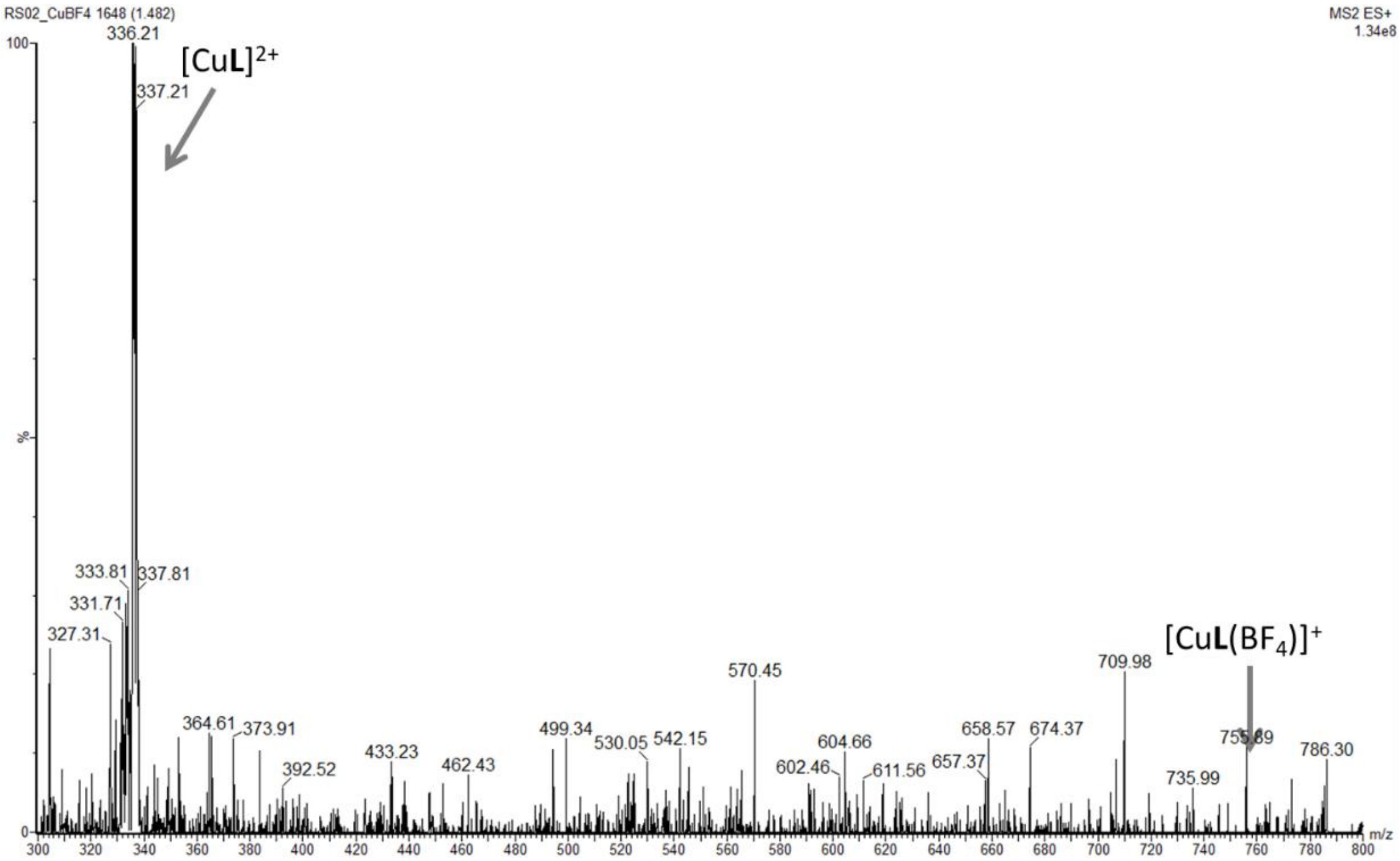

Figure S6: ESI-MS of **1-Cu** in CH_3CN.

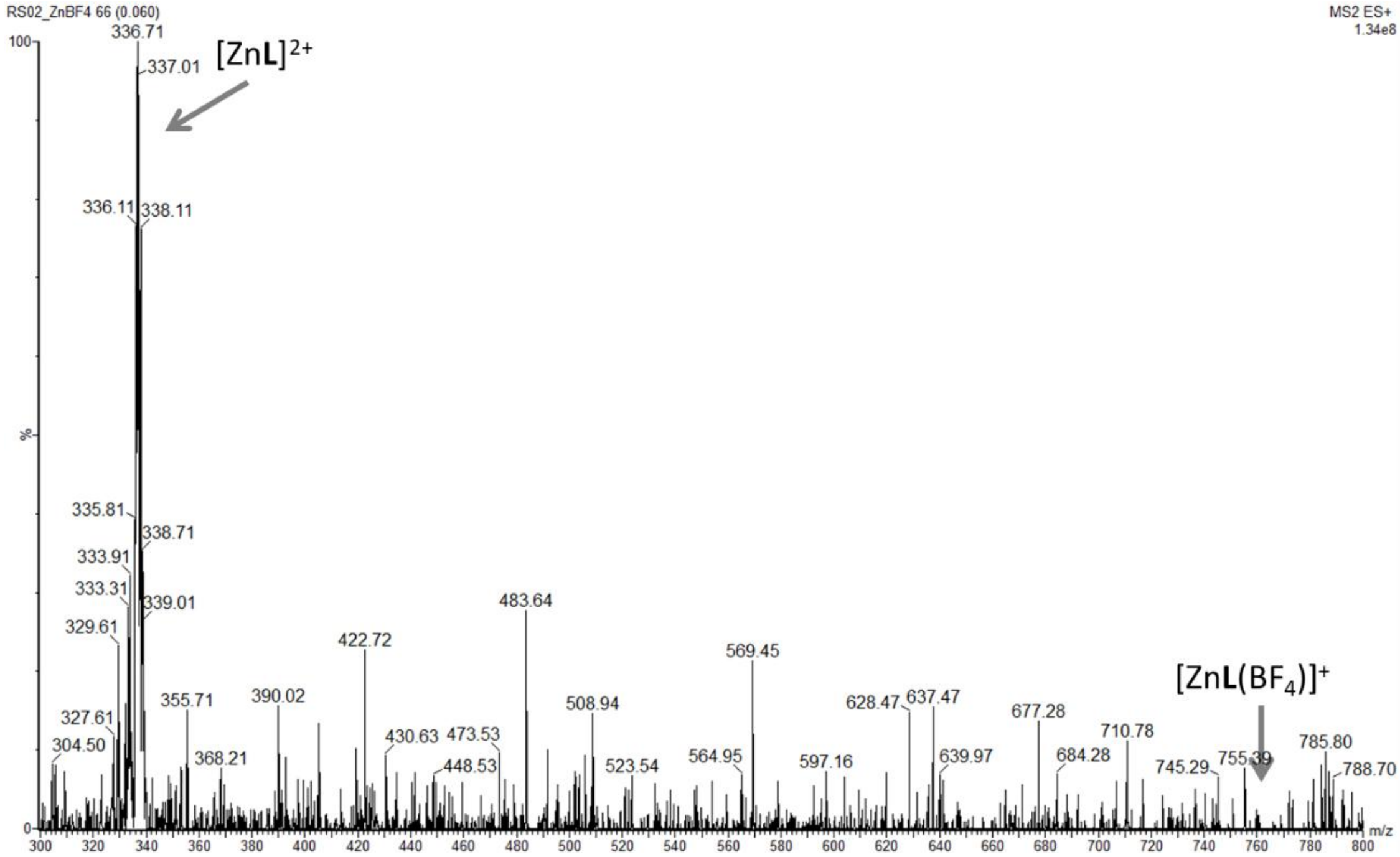

Figure S7: ESI-MS of **1-Zn** in CH_3CN.

NMR

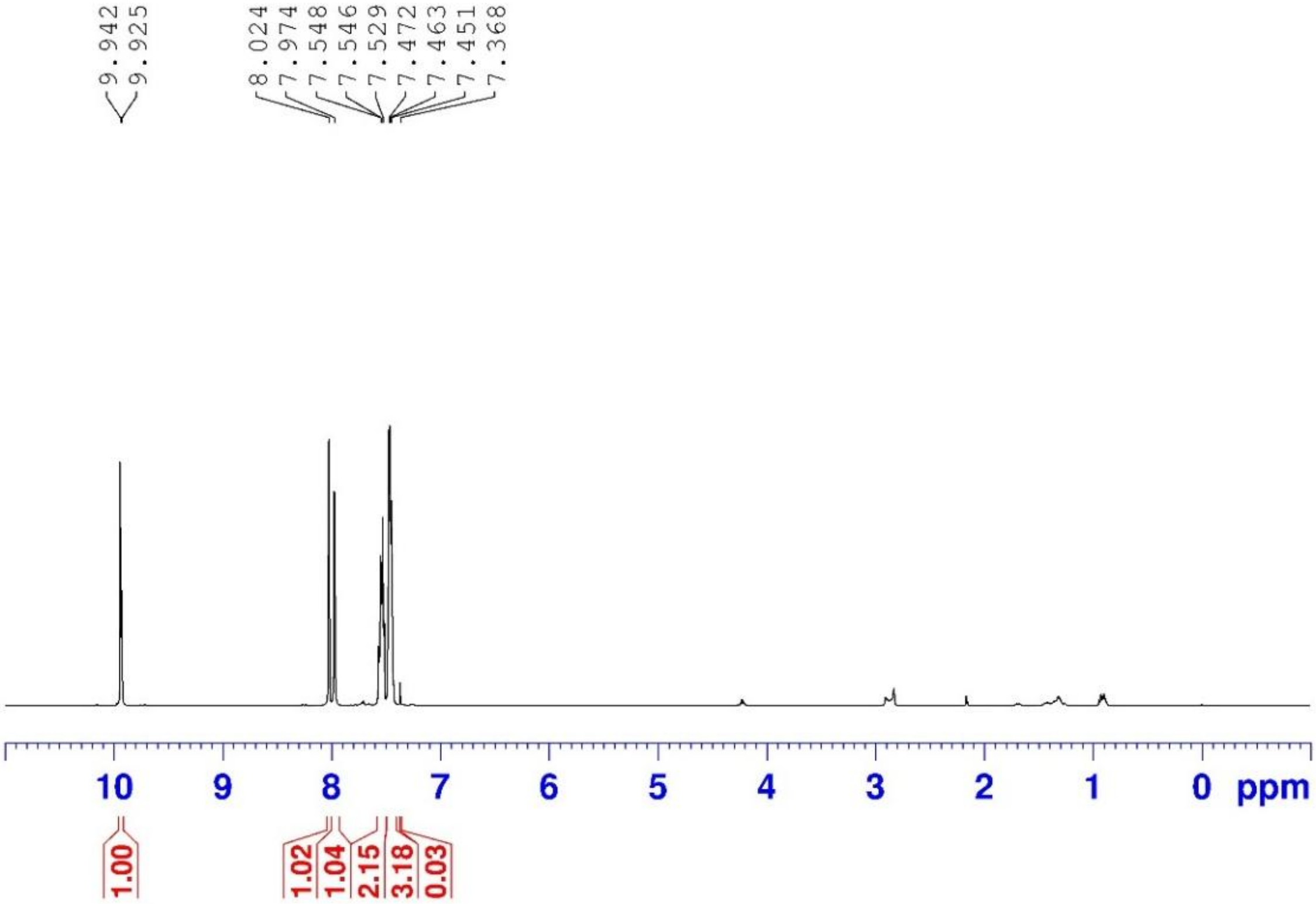

Figure S8: ^{1}H NMR spectrum of 1-(phenyl)-1*H*-imidazole-4-carbaldehyde in $CDCl_3$.

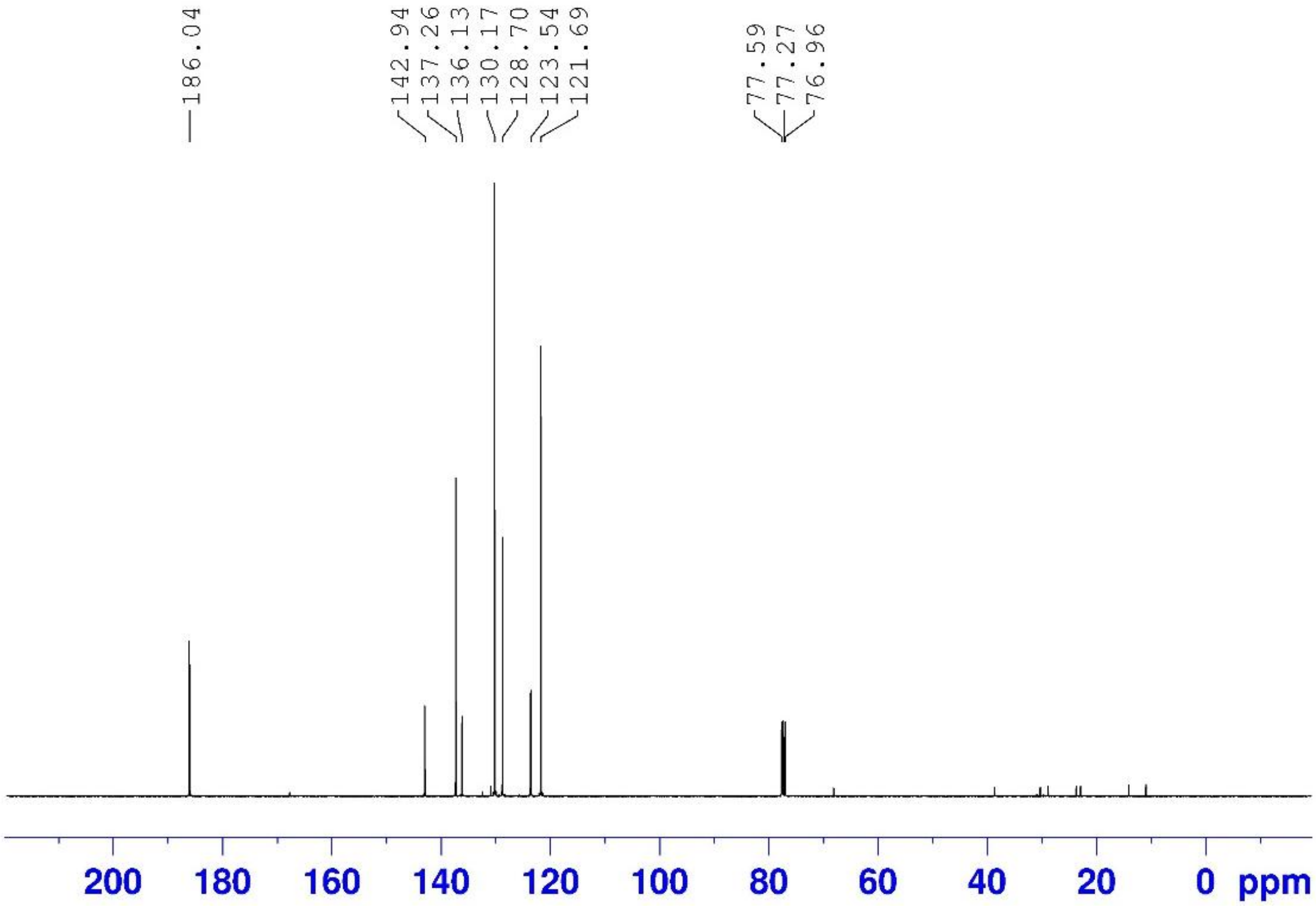

Figure S9: ^{13}C NMR spectrum of 1-(phenyl)-1*H*-imidazole-4-carbaldehyde in $CDCl_3$.

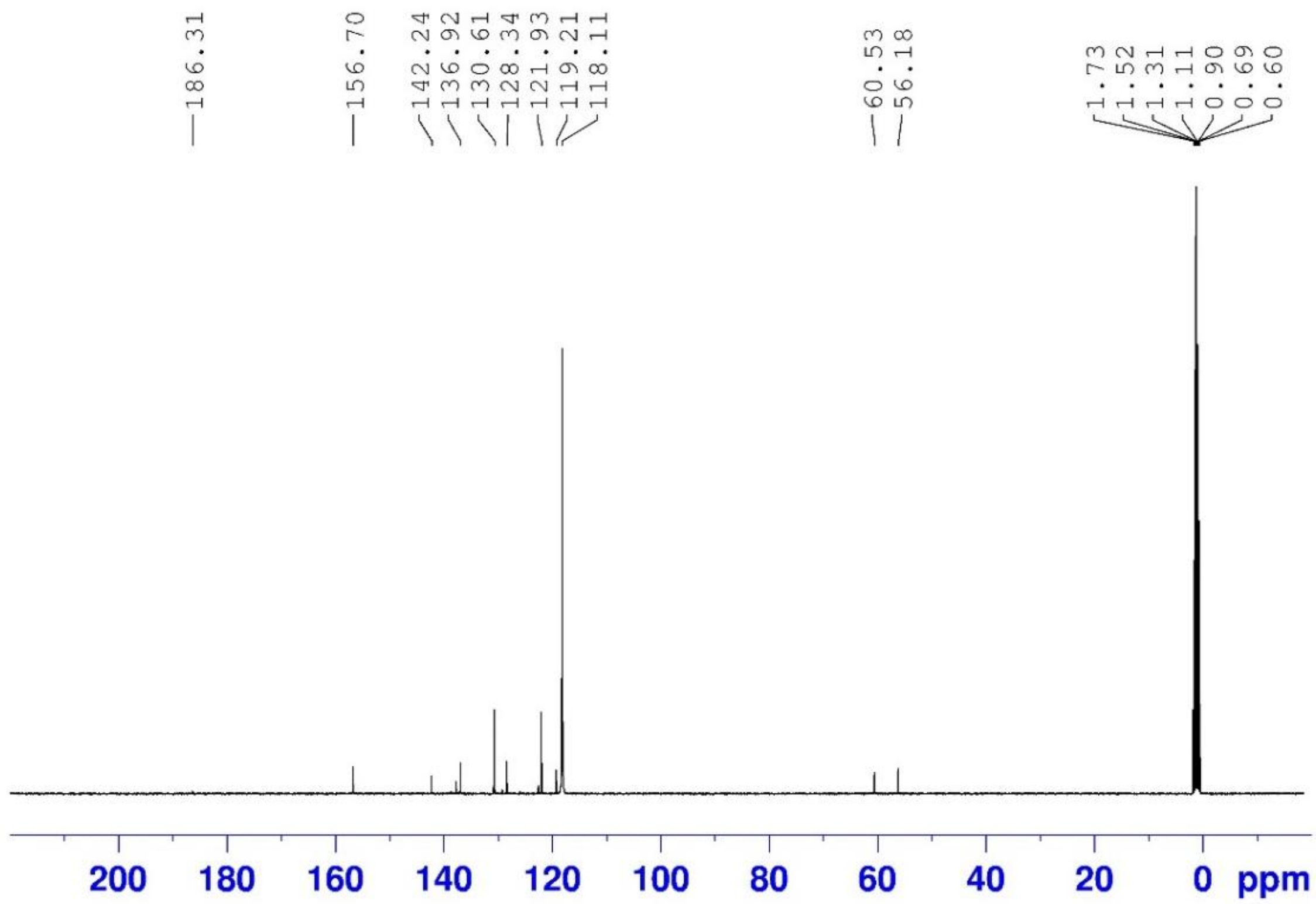

Figure S10: ^{13}C NMR spectrum of crude **L** in CD_3CN.

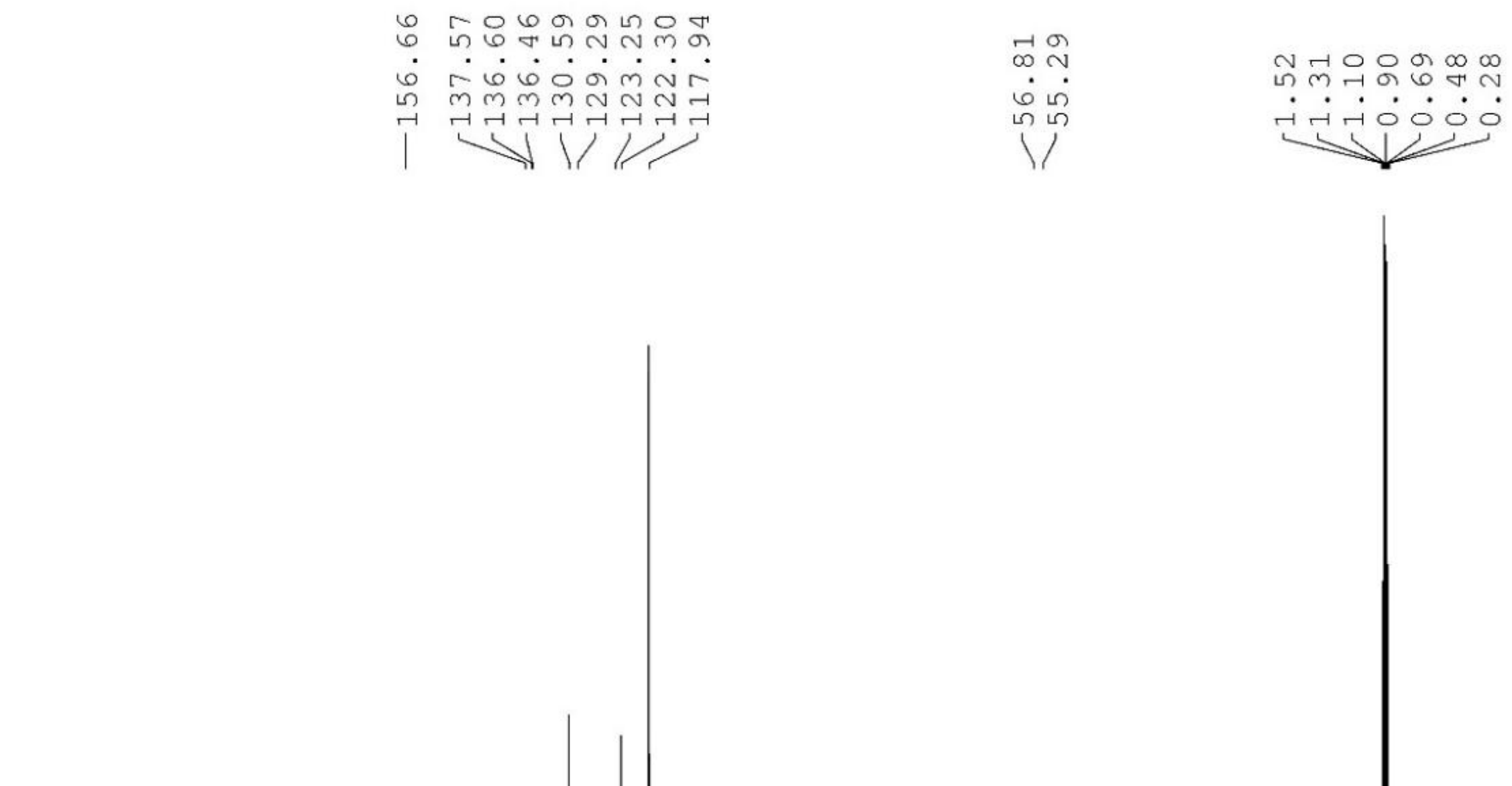

Figure S11: ^{13}C NMR spectrum of (**1-Zn**) in CD_3CN.

FT-IR

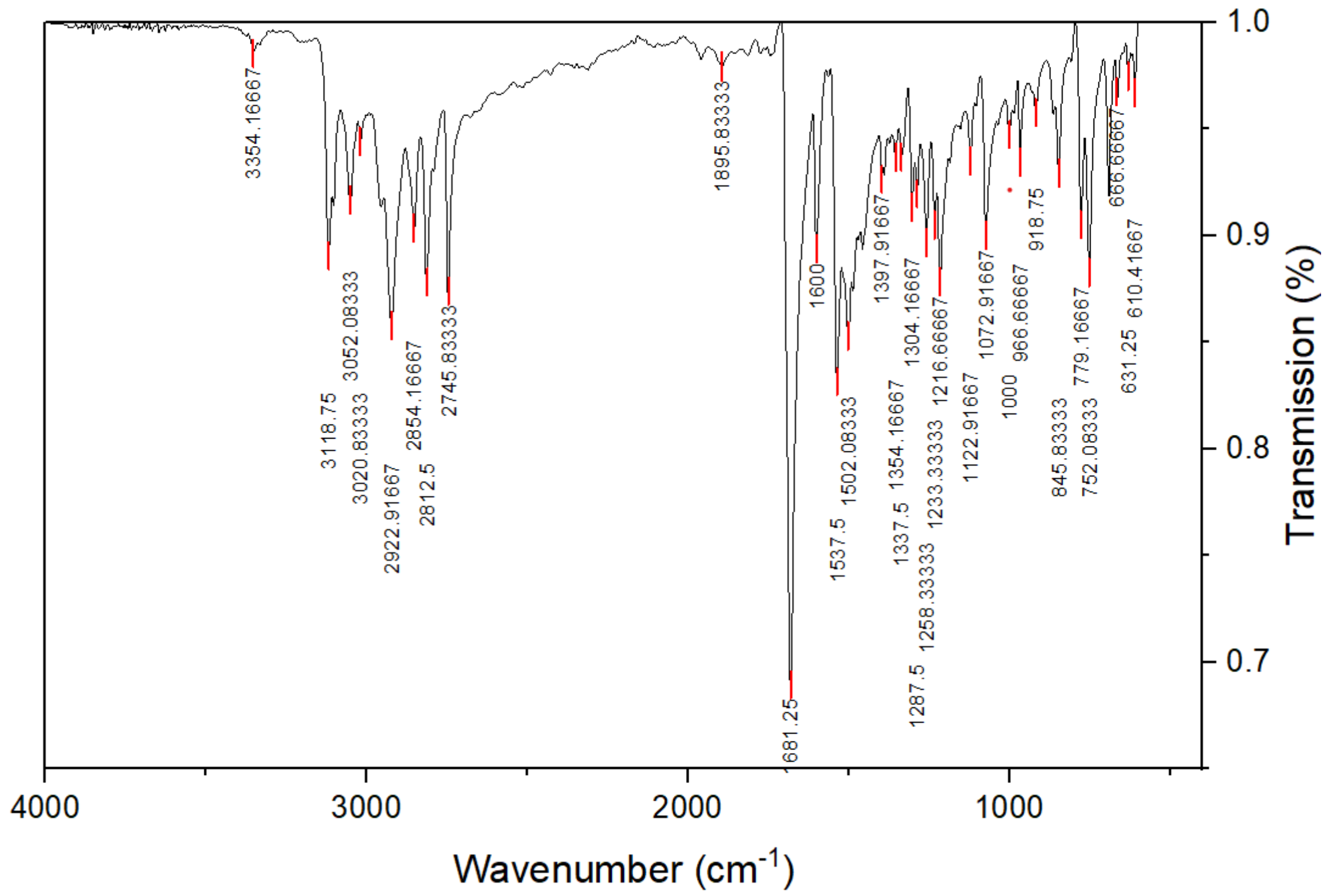

Figure S12: FT-IR spectrum of 1-(phenyl)-1*H*-imidazole-4-carbaldehyde

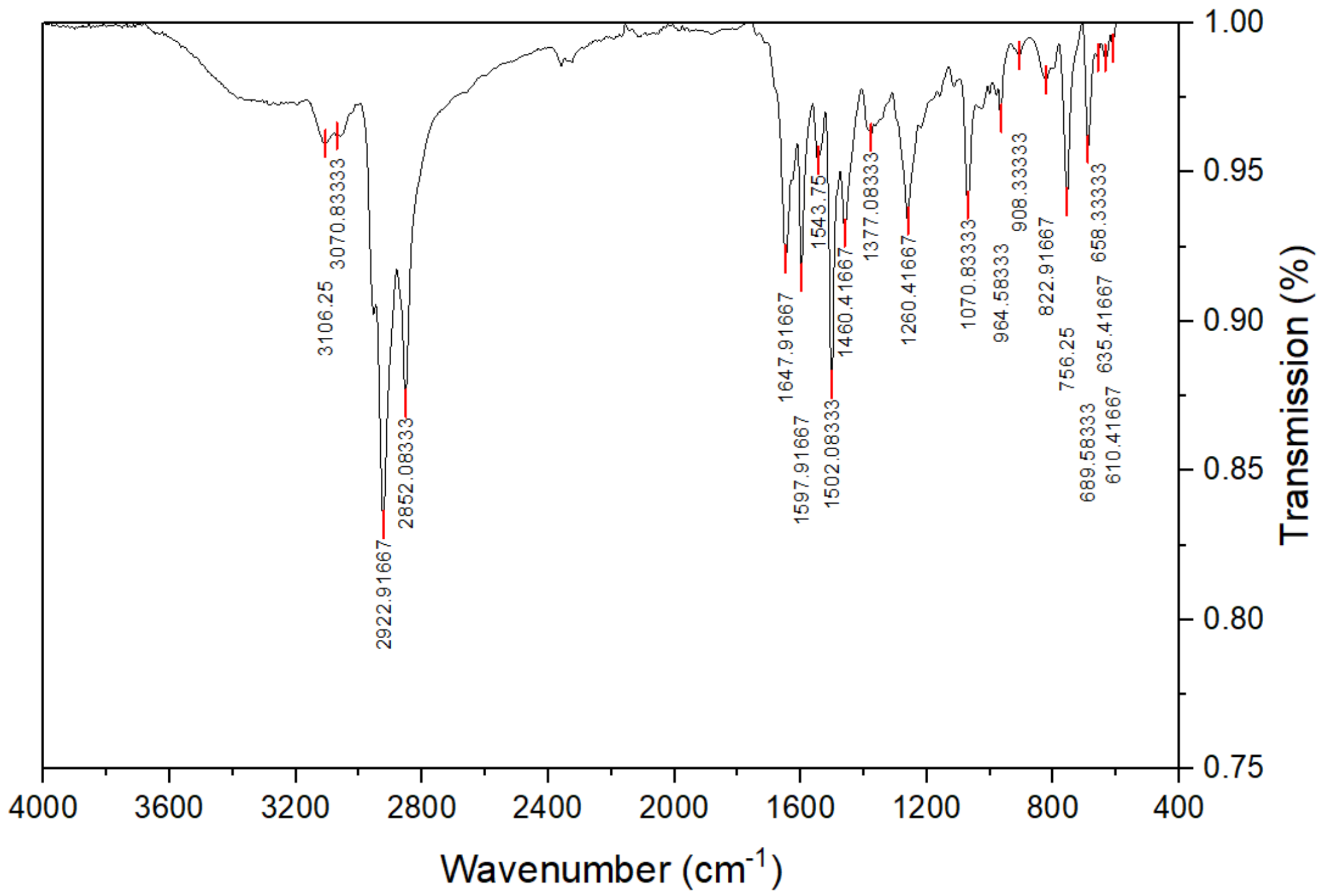

Figure S13: FT-IR spectrum of **L**

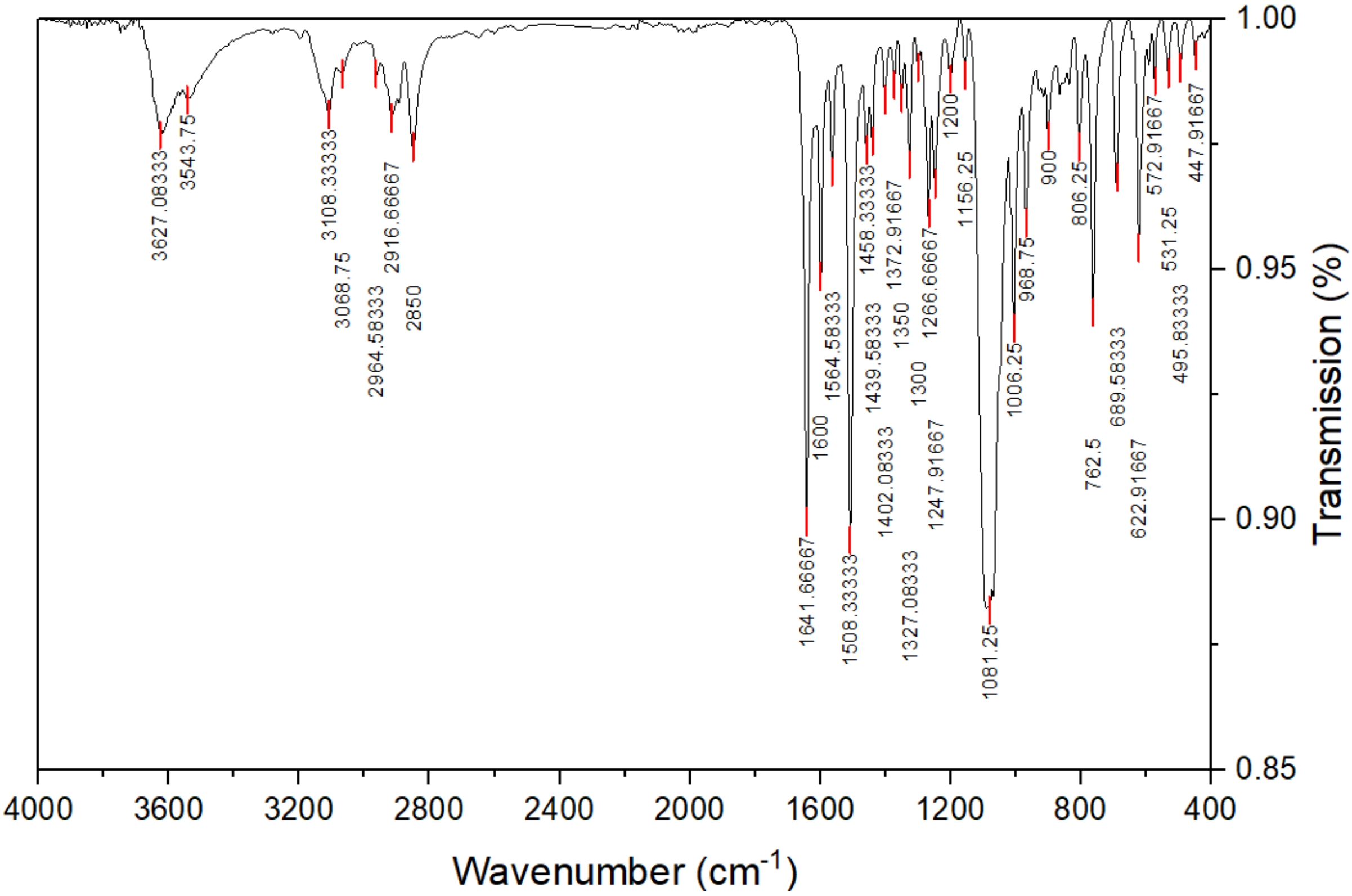

Figure S14: FT-IR spectrum of **1-Mn**

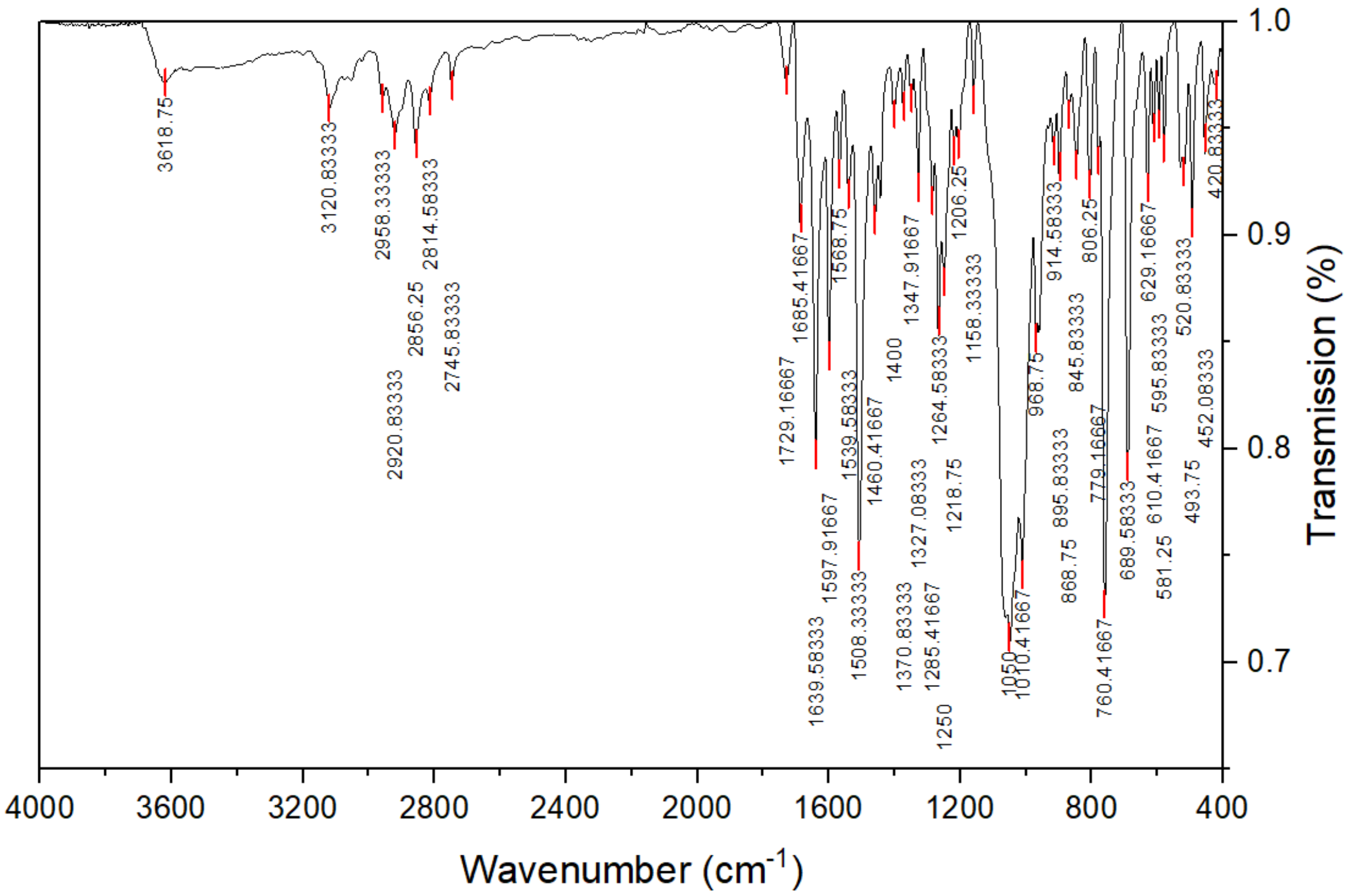

Figure S15: FT-IR spectrum of **1-Co**

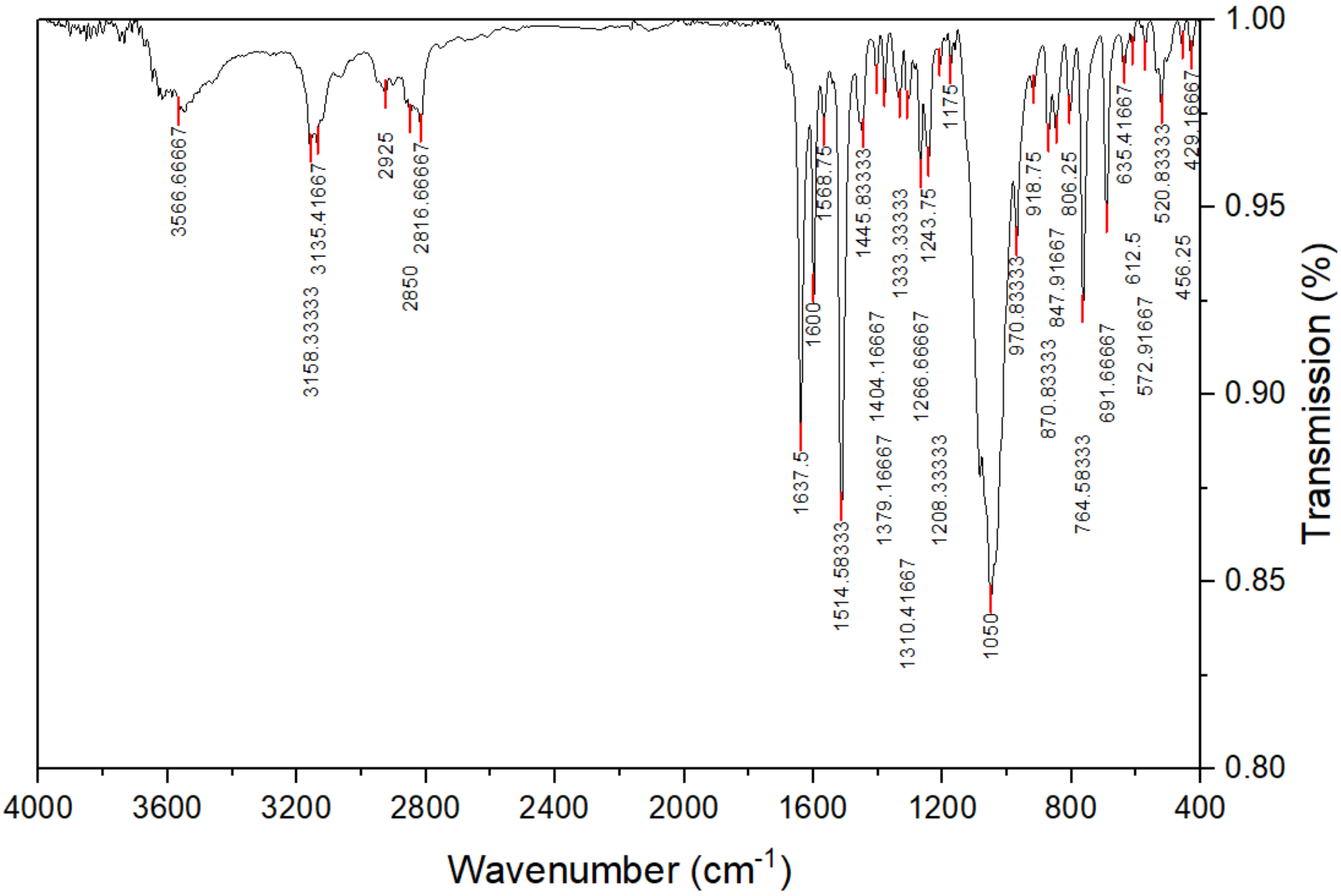

Figure S16: FT-IR spectrum of **1-Ni**

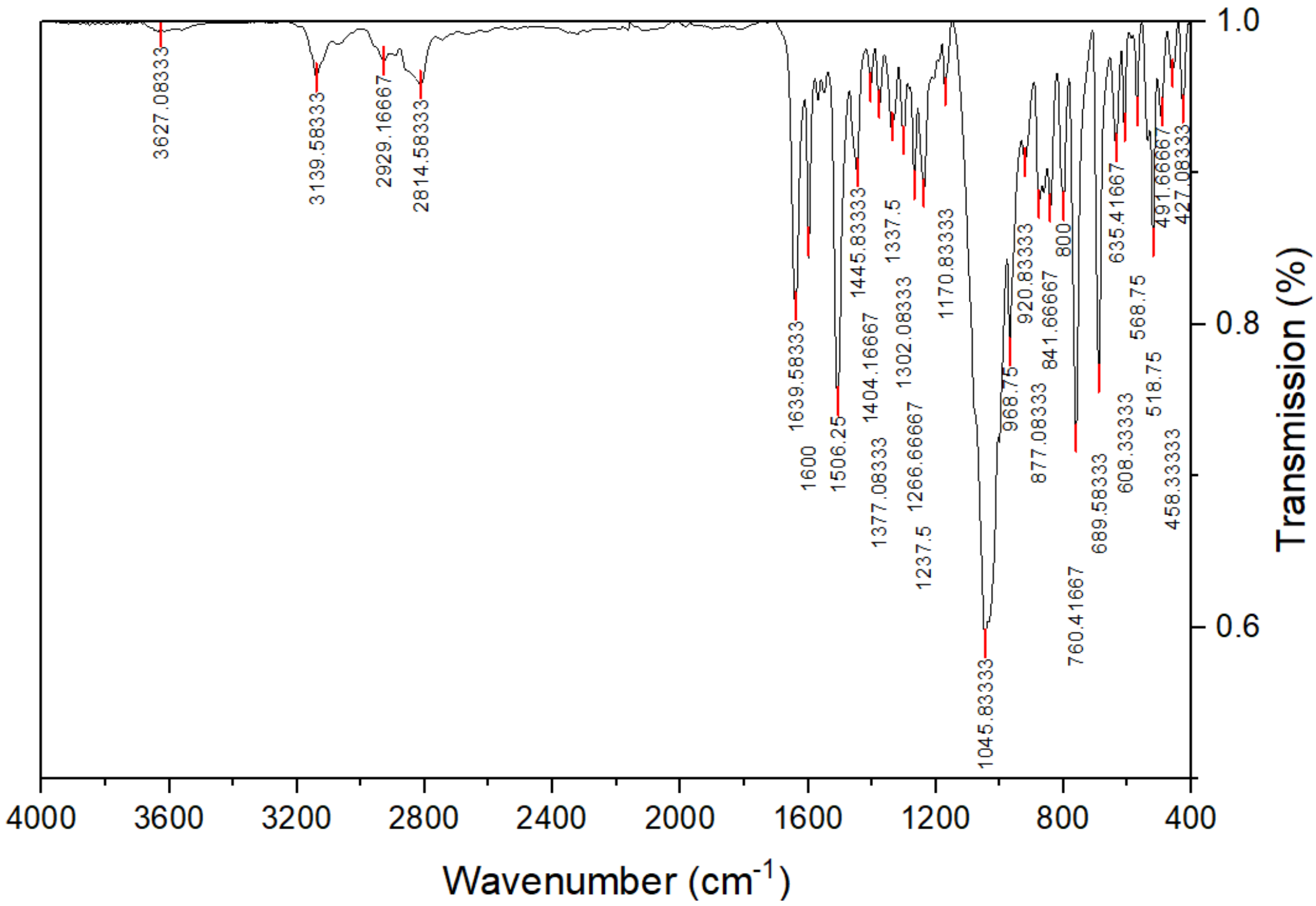

Figure S17: FT-IR spectrum of **1-Cu**

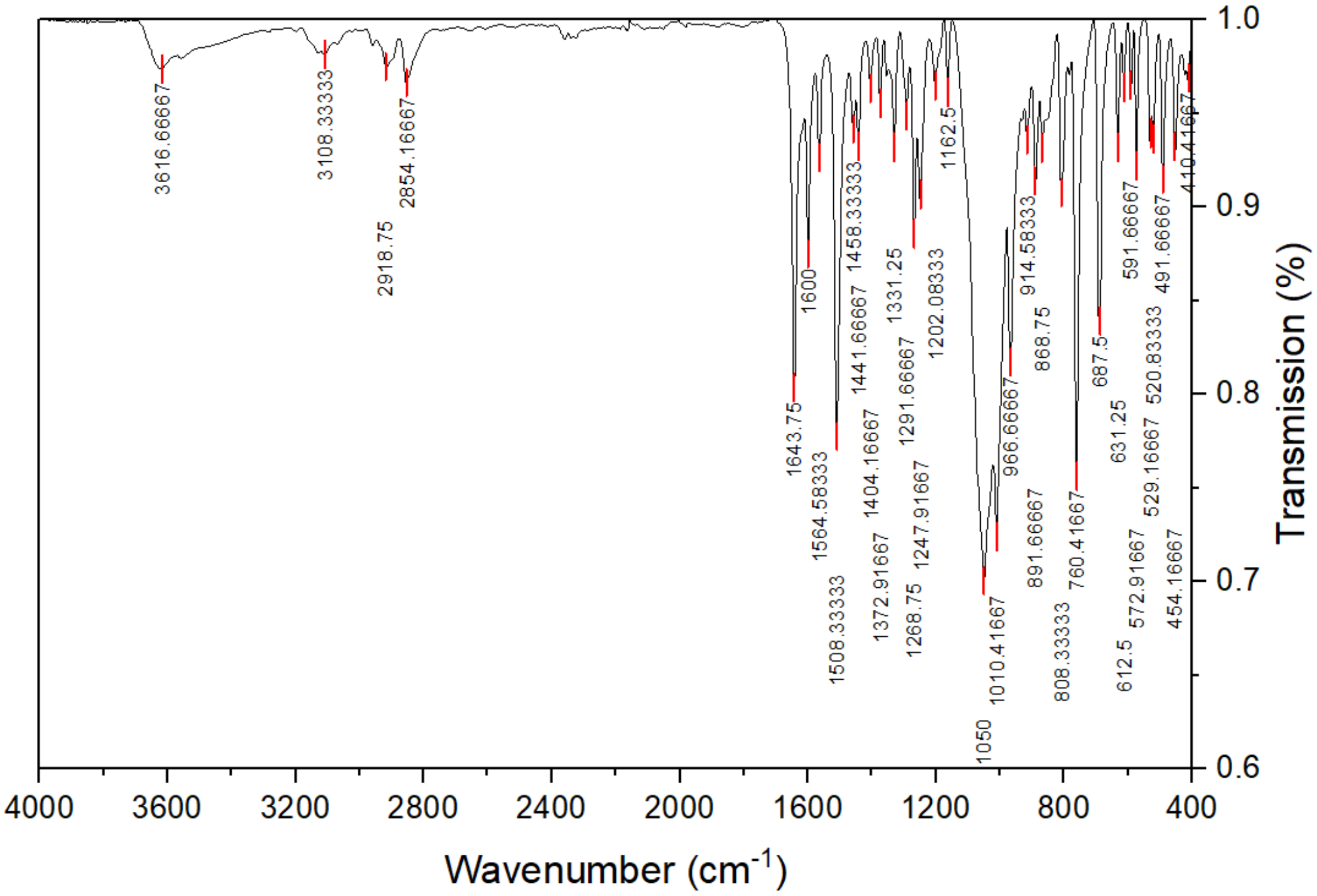

Figure S18: FT-IR spectrum of **1-Zn**

SEM-EDS

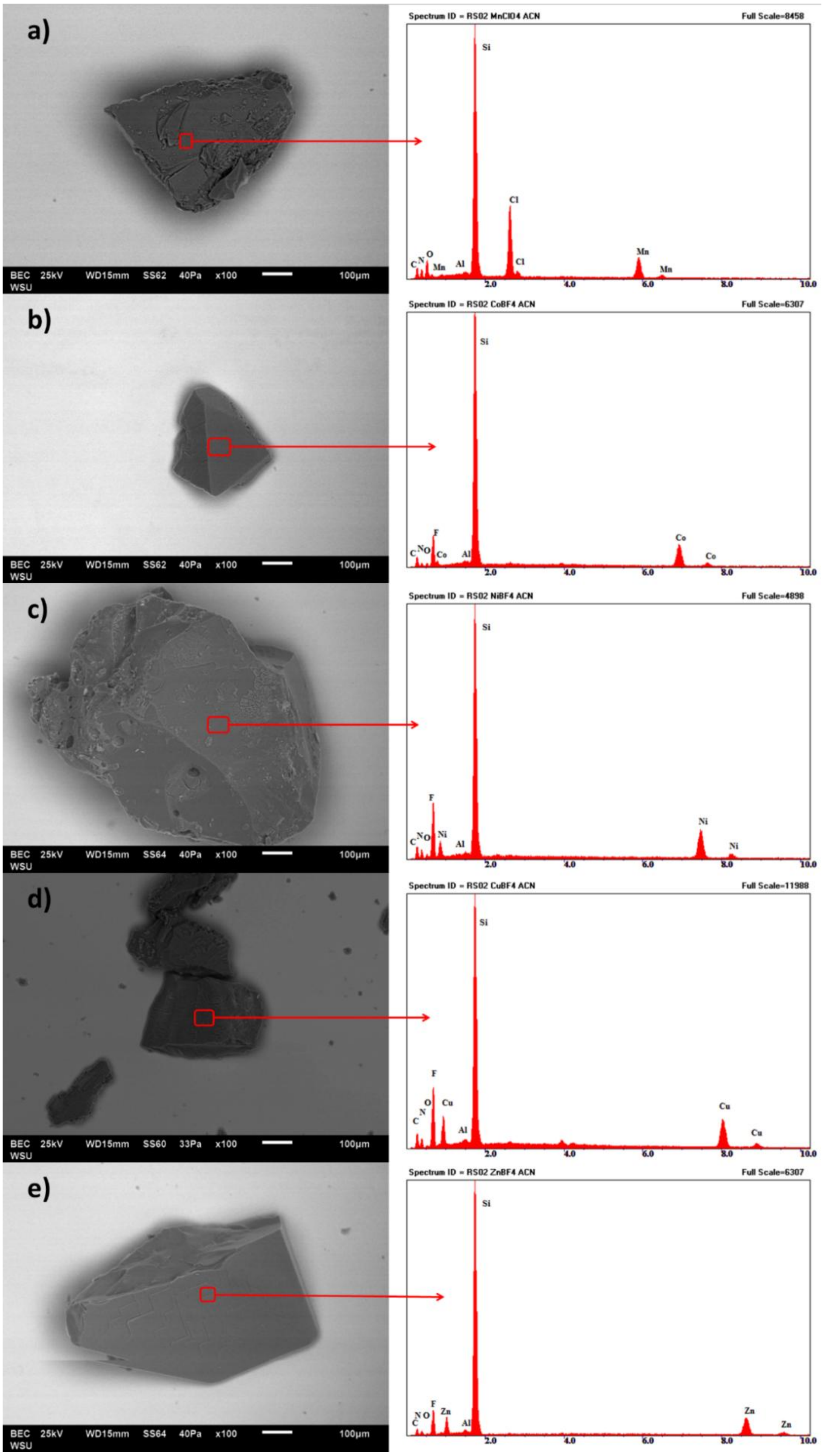

Figure S19: A backscattering SEM image (left) and corresponding EDS analysis (right) for a) **1-Mn**, b) **1-Co**, c) **1-Ni**, d) **1-Cu** and e) **1-Zn**

Optical Microscopy

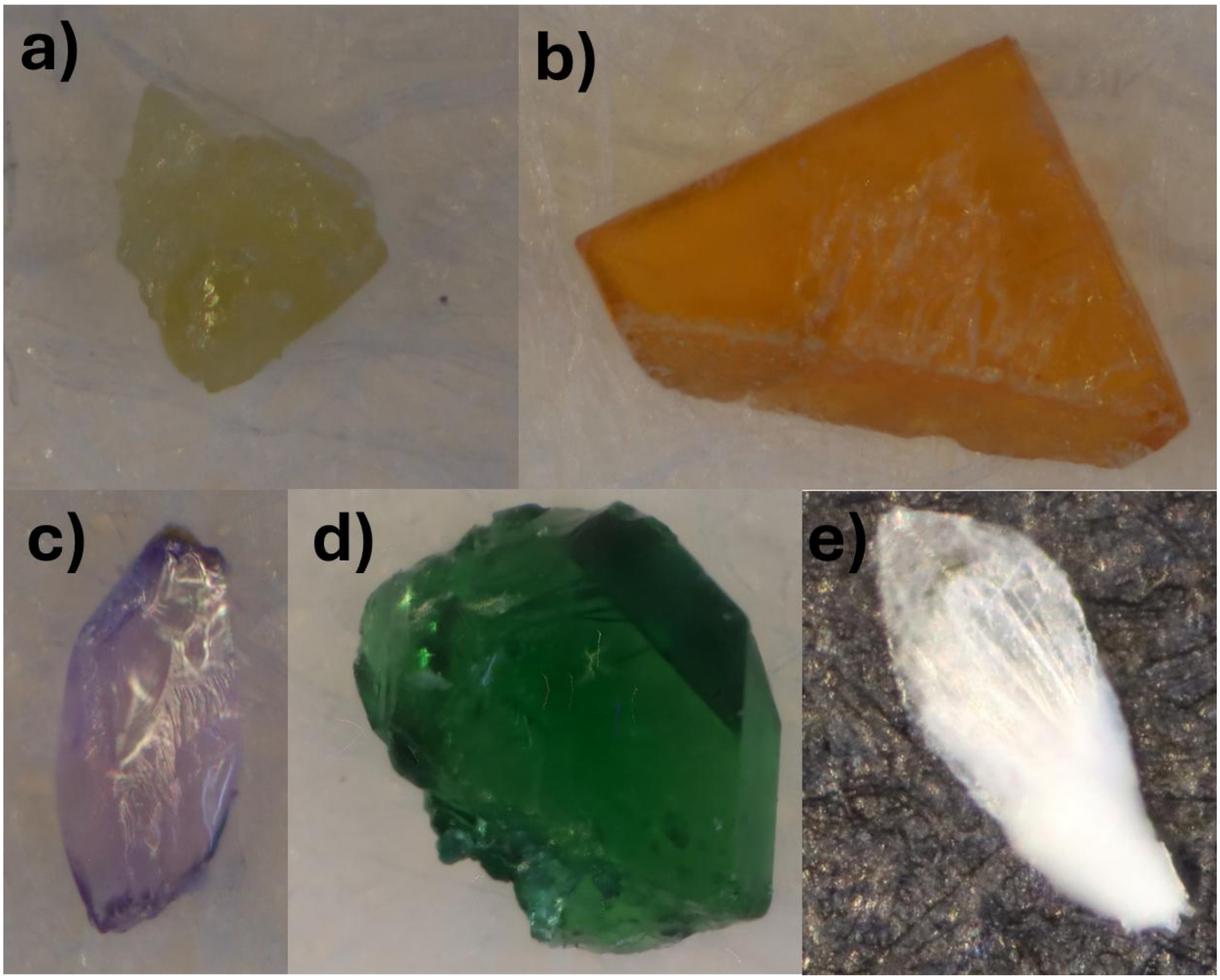

Figure S20: An optical microscope image for a) **1-Mn**, b) **1-Co**, c) **1-Ni**, d) **1-Cu** and e) **1-Zn**

UV-Vis

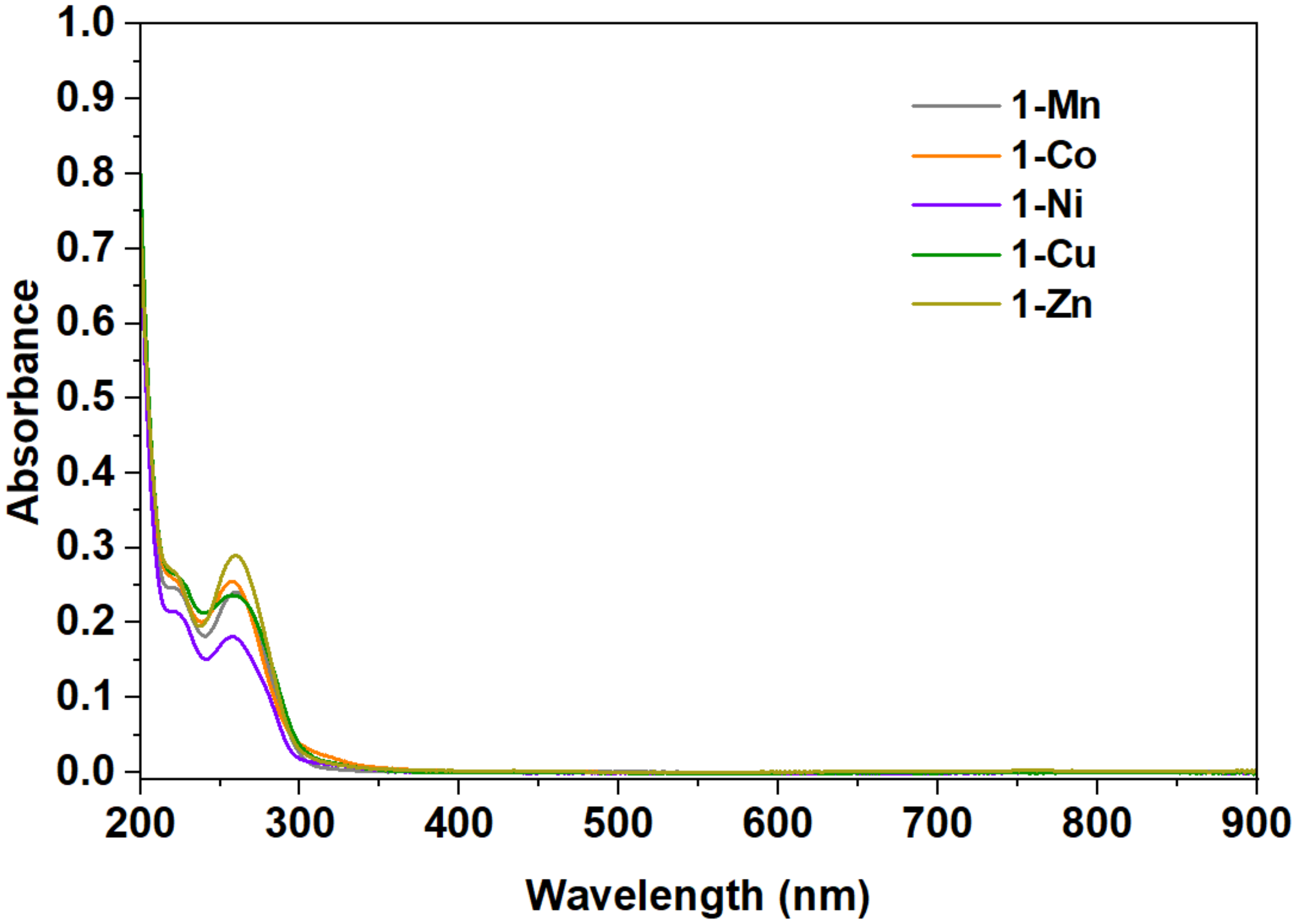

Figure S21: UV-Vis spectra from 200-900 nm of dilute solutions of **1-Mn**, **1-Co**, **1-Ni**, **1-Cu** and **1-Zn**.

STA

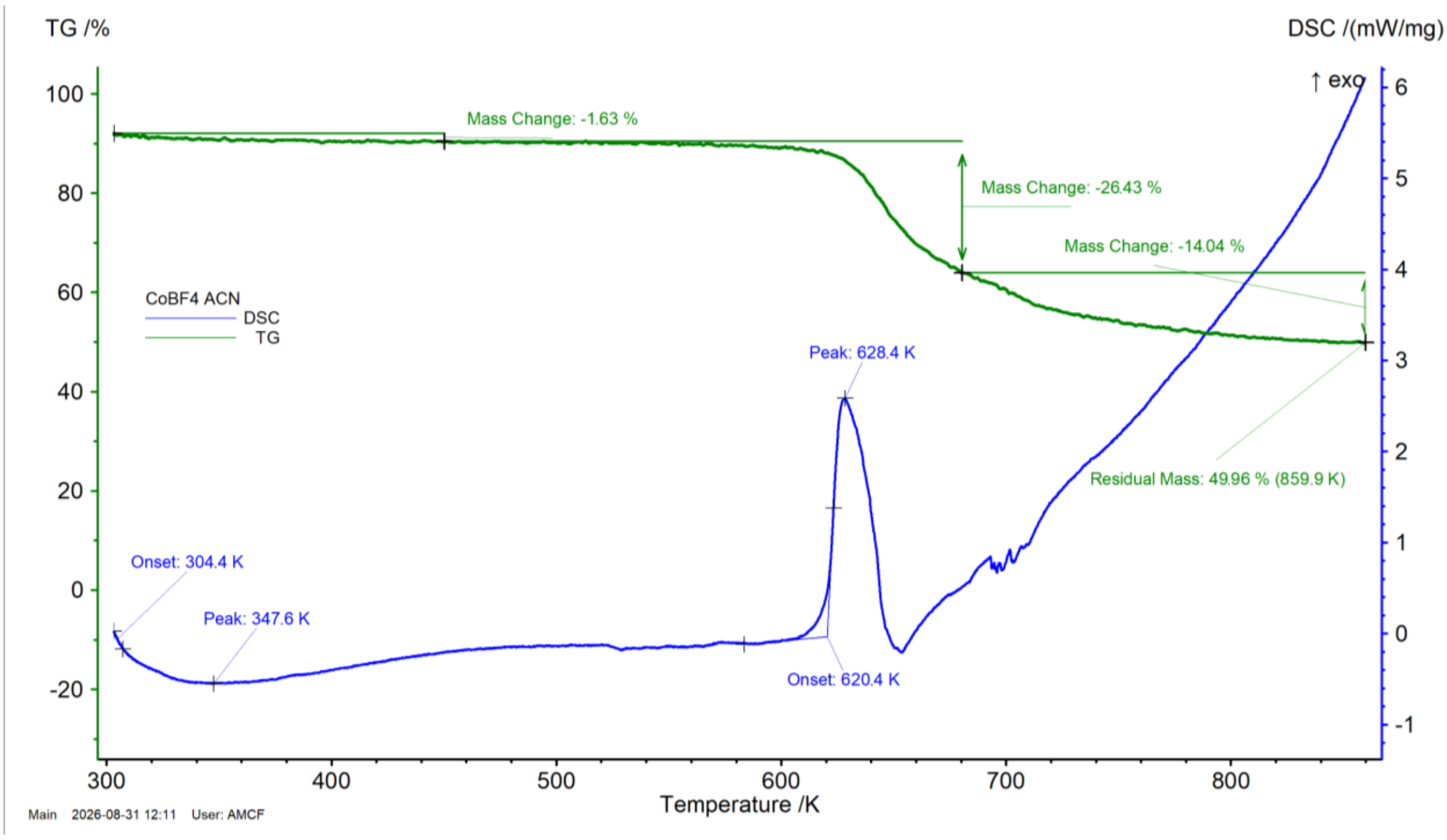

Figure S22: STA of **1-Co**

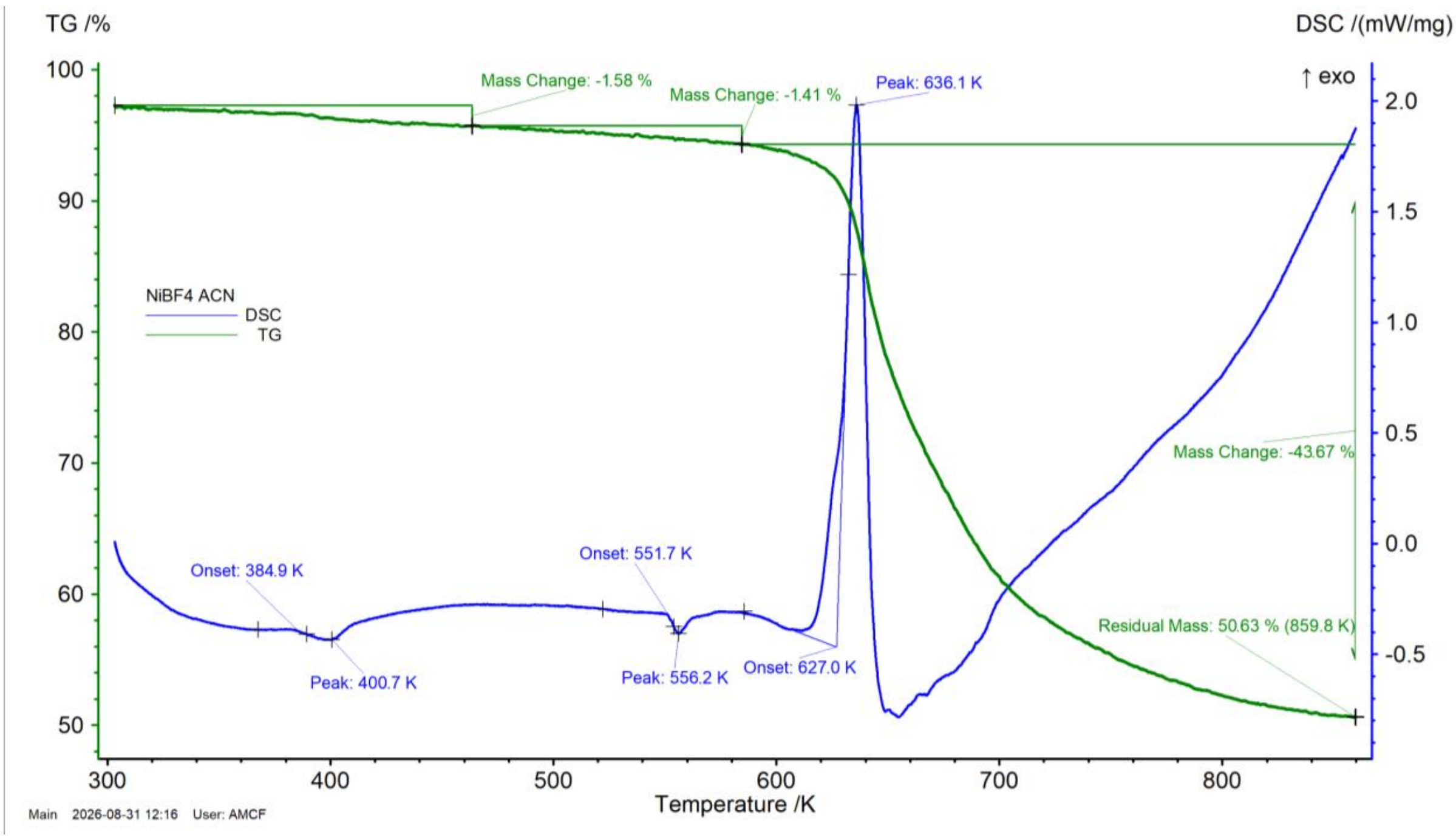

Figure S23: STA of **1-Ni**

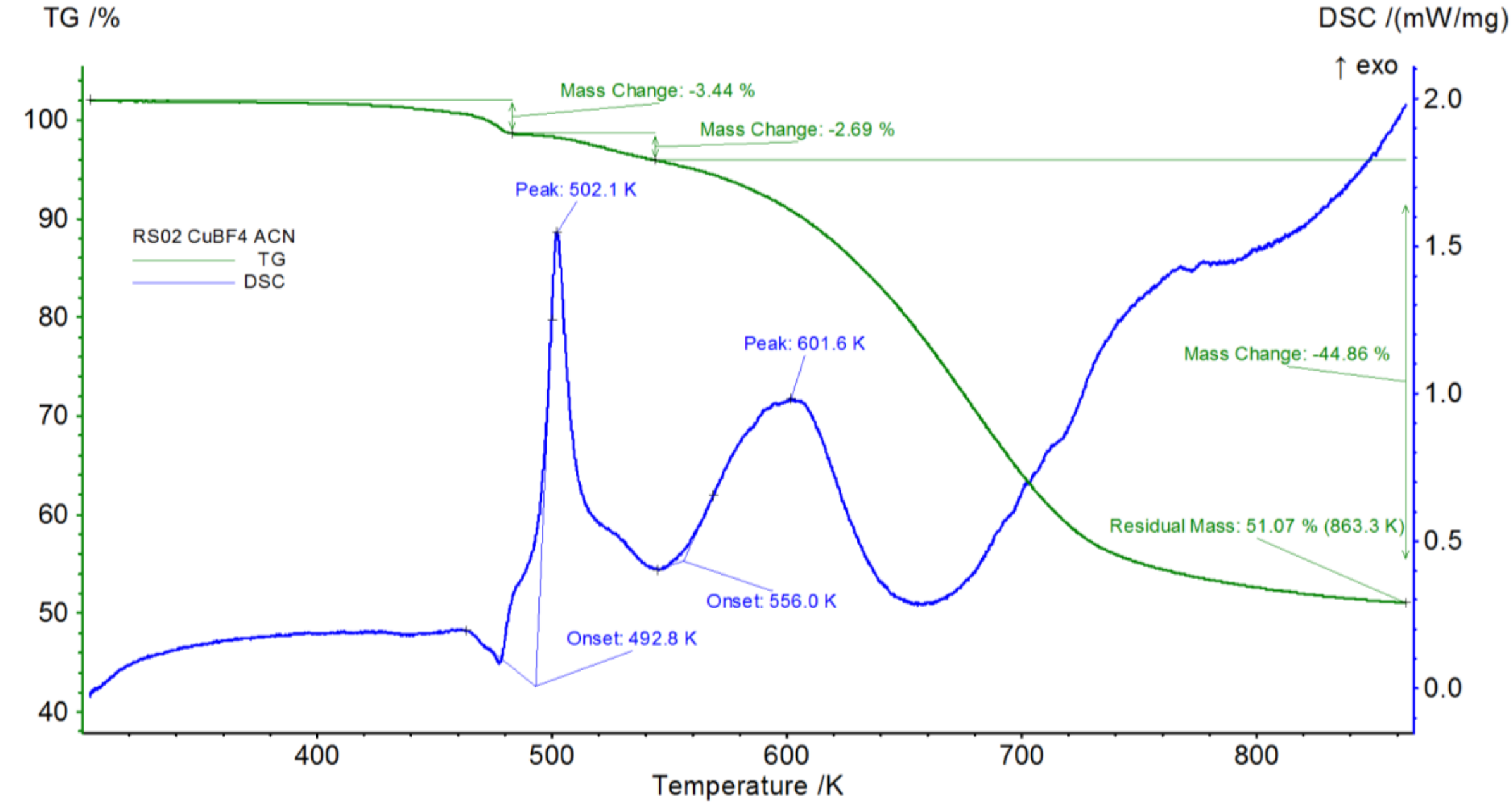

Figure S24: STA of **1-Cu**

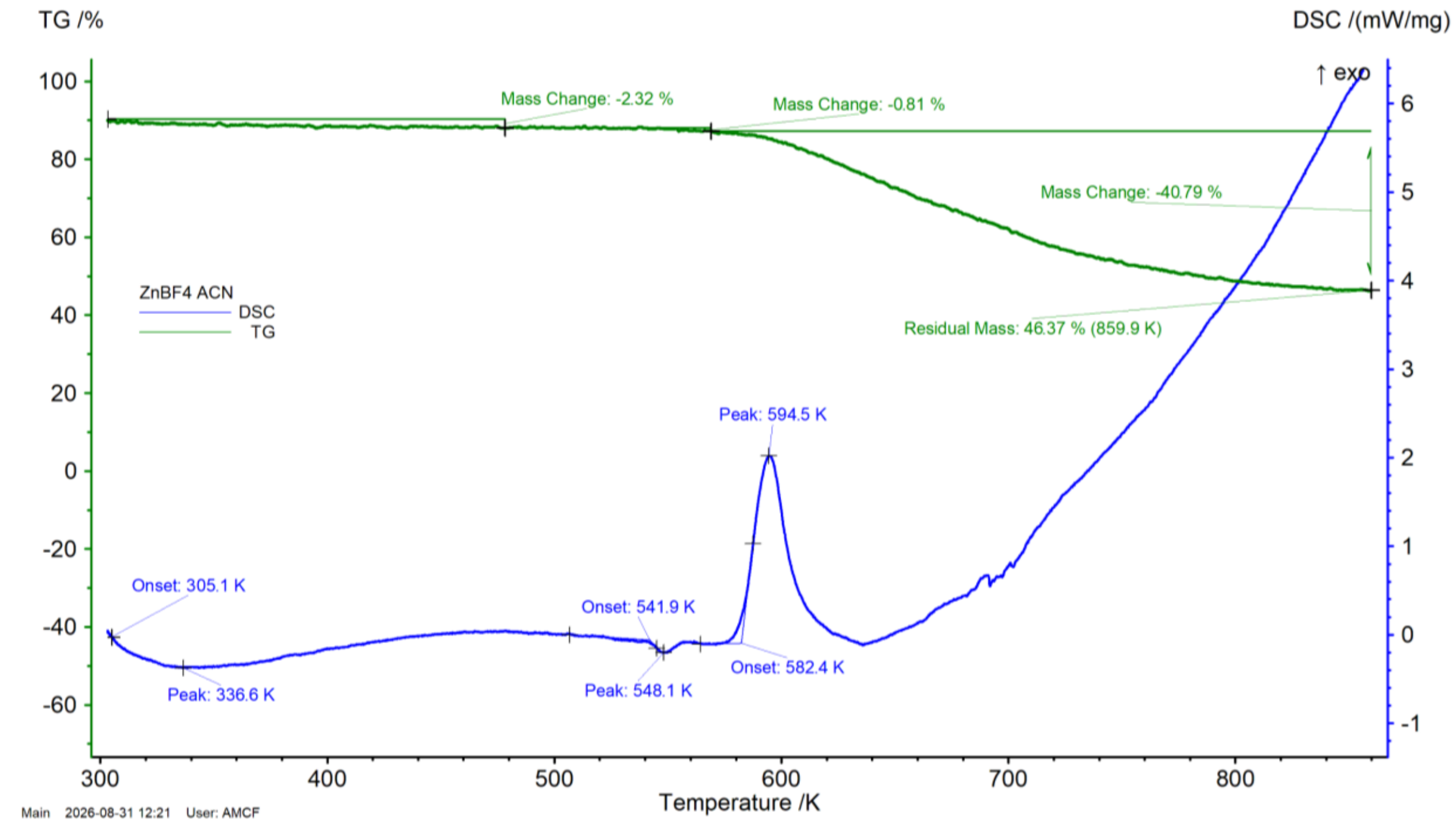

Figure S25: STA of **1-Zn**

PXRD

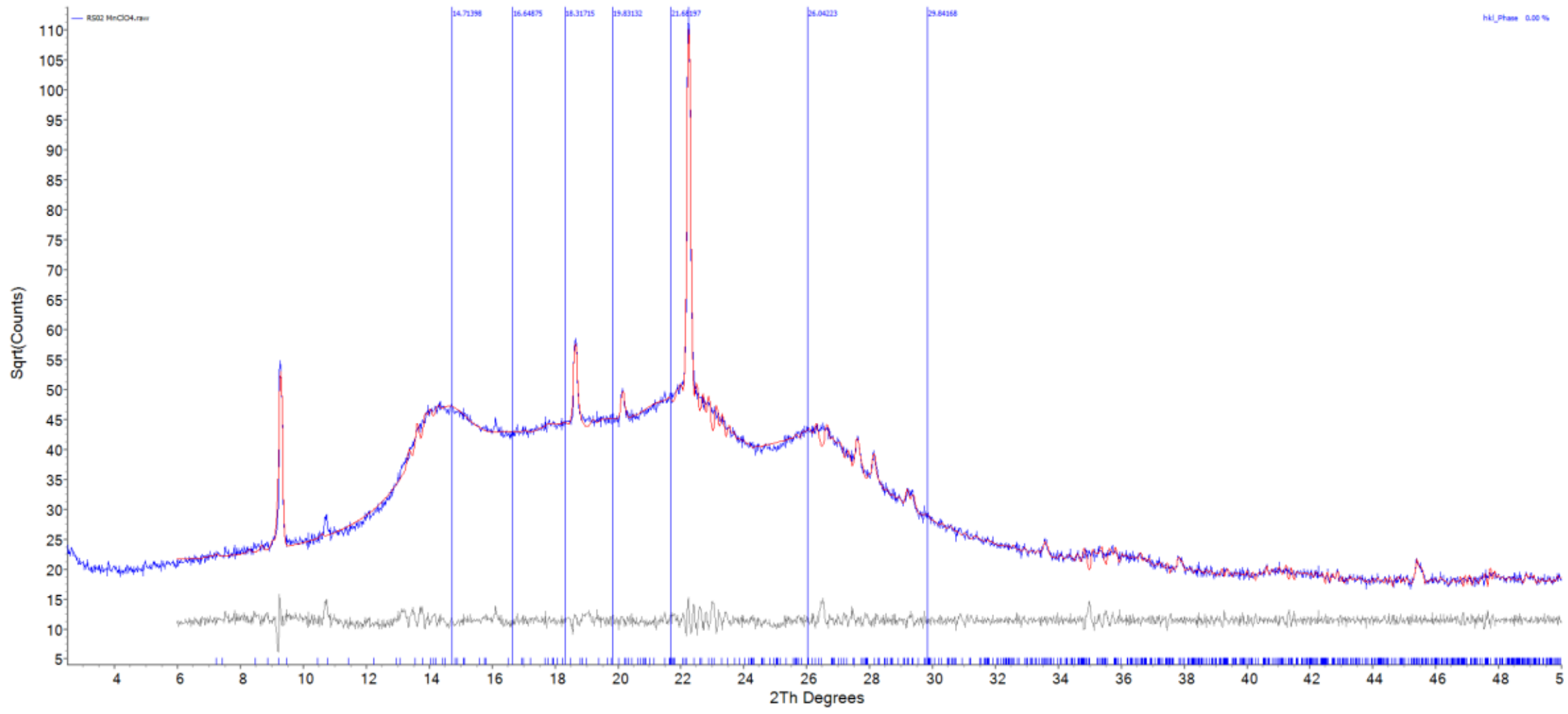

Figure S26: Le Bail refinement of the simulated (red) and experimental (blue) powder pattern with the difference (grey) of **1-Mn** obtained at room temperature. Blue lines are attributed to manually placed peak maxima associated with the superposition of the amorphous Kapton foil pattern. Rexp = 3.08, Rwp = 4.51, Rp = 3.32, GOF = 1.47

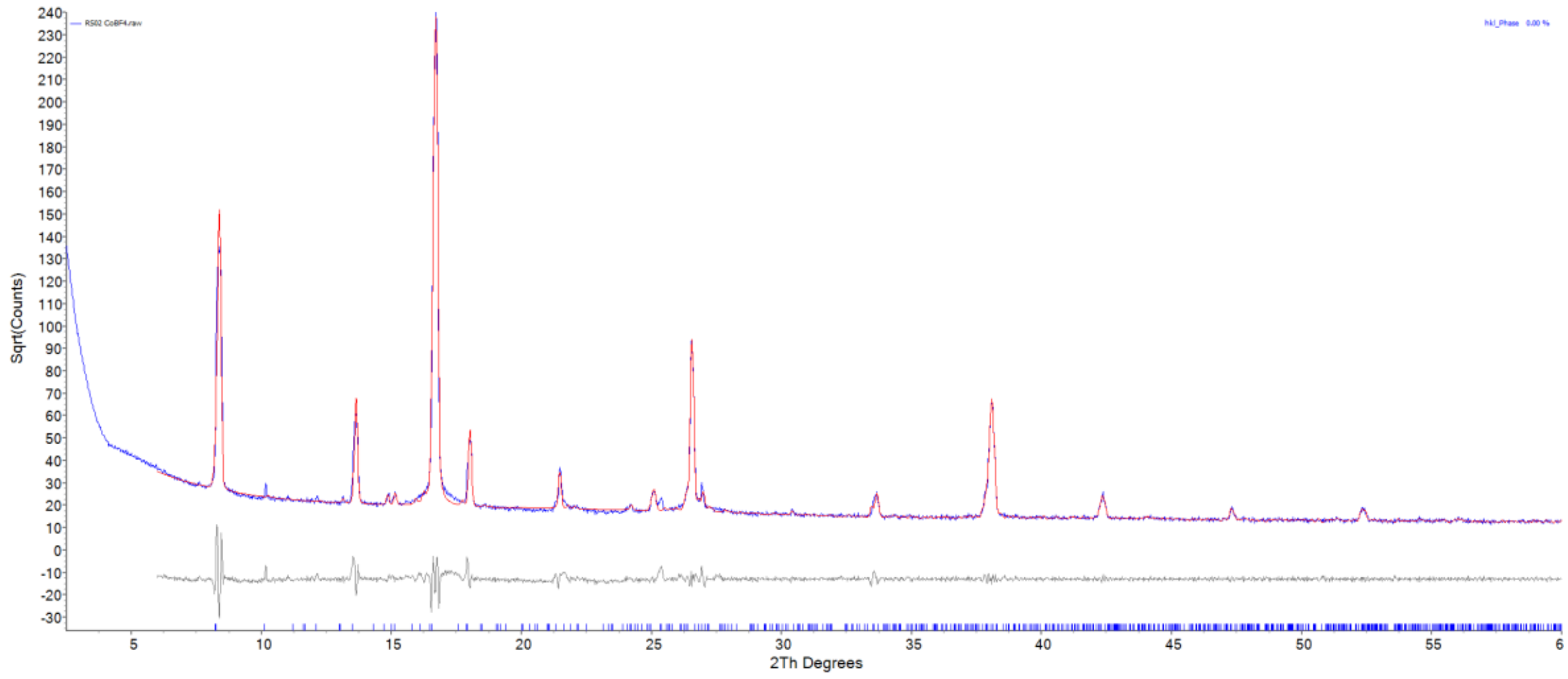

Figure S27: Le Bail refinement of the simulated (red) and experimental (blue) powder pattern with the difference (grey) of **1-Co** obtained at room temperature. Rexp = 3.97, Rwp = 13.18, Rp = 9.00, GOF = 3.32

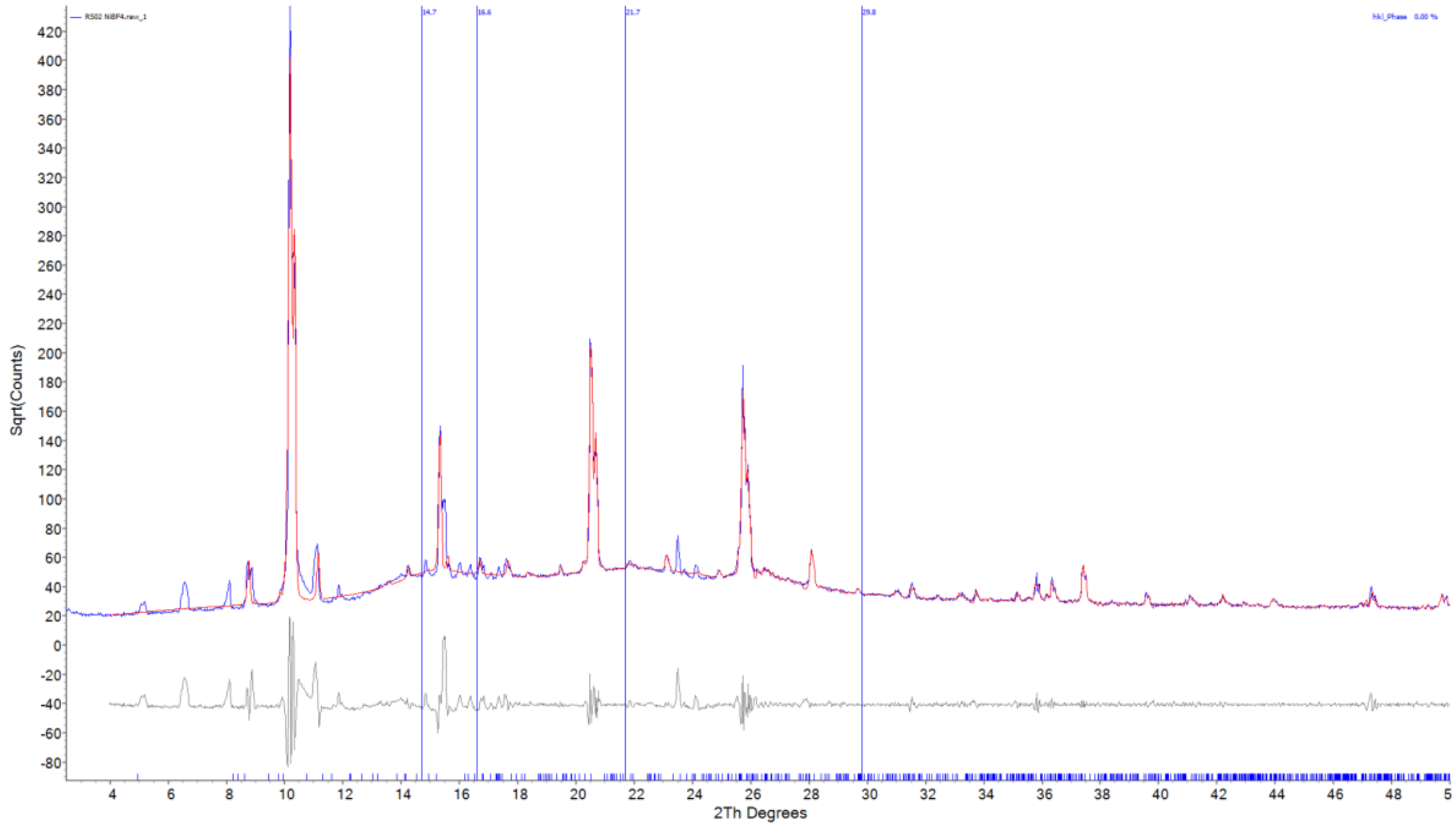

Figure S28: Le Bail refinement of the simulated (red) and experimental (blue) powder pattern with the difference (grey) of **1-Ni** obtained at room temperature. Blue lines are attributed to manually placed peak maxima associated with the superposition of the amorphous Kapton foil pattern. Rexp = 2.07, Rwp = 21.03, Rp = 13.53, GOF = 10.16

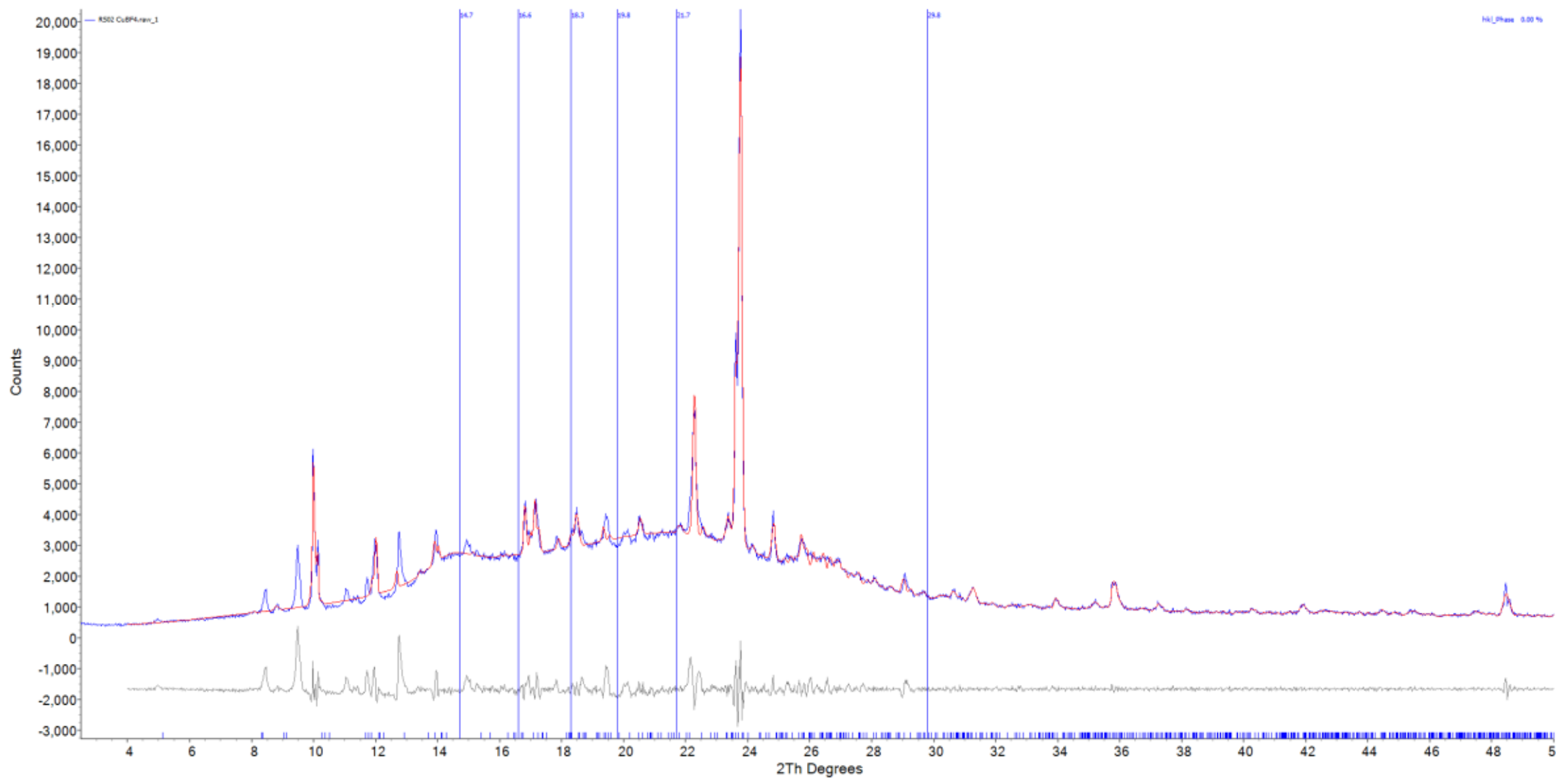

Figure S29: Le Bail refinement of the simulated (red) and experimental (blue) powder pattern with the difference (grey) of **1-Cu** obtained at room temperature. Blue lines are attributed to manually placed peak maxima associated with the superposition of the amorphous Kapton foil pattern. Rexp = 2.40, Rwp = 8.14, Rp = 4.80, GOF = 3.39

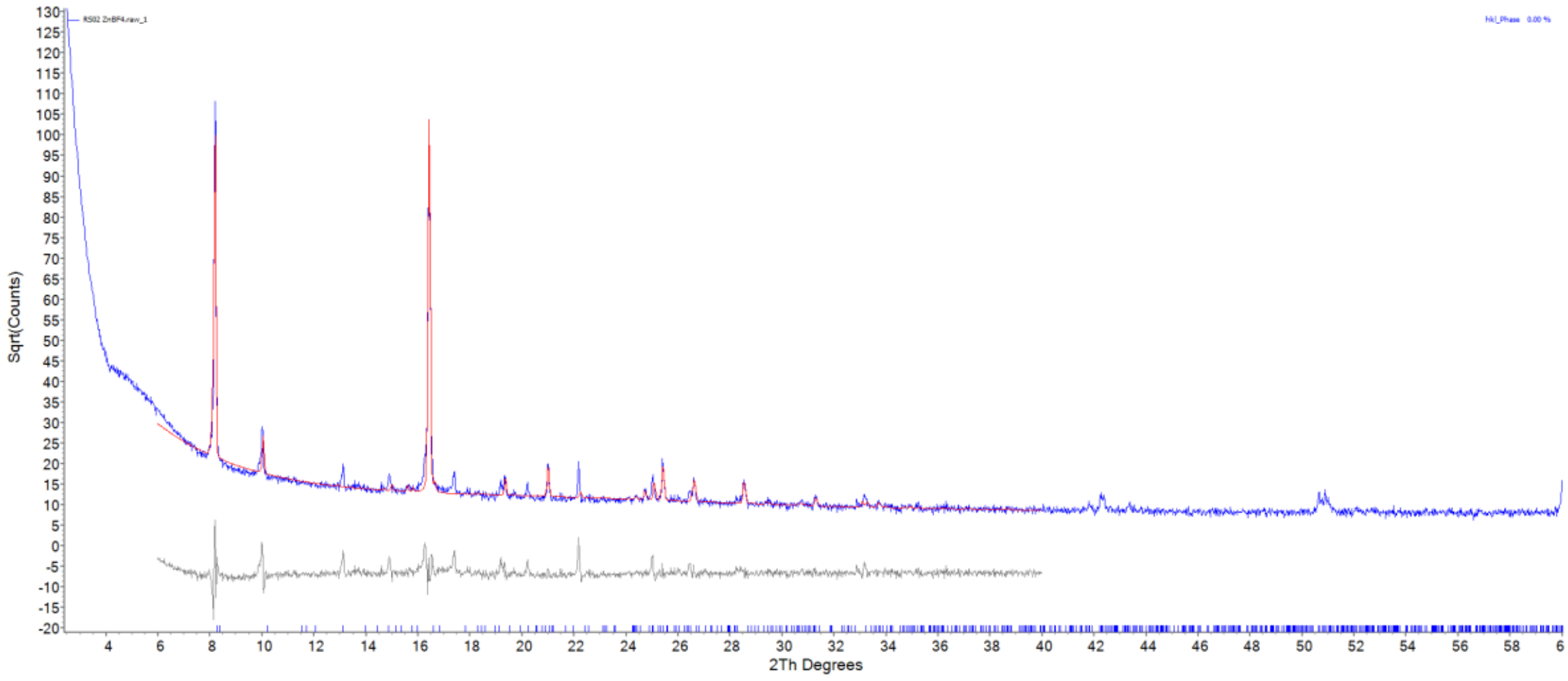

Figure S30: Le Bail refinement of the simulated (red) and experimental (blue) powder pattern with the difference (grey) of **1-Zn** obtained at room temperature. Rexp = 7.47, Rwp = 18.10, Rp = 13.25, GOF = 2.42

Crystallographic Information

Table S1: Crystallographic data table of all metal complexes.

Compound	**1-Mn**	**1-Co**	**1-Ni**	**1-Cu**	**1-Zn**
CCDC Number	2565199	2565197	2565196	2565200	2565198
Empirical formula	$C_{38}H_{41}Cl_2MnN_{11}O_9$	$C_{36}H_{36}B_2CoF_8N_{10}$	$C_{37}H_{37.5}B_2F_8N_{10.5}NiO_{0.5}$	$C_{38}H_{39}B_2CuF_8N_{11}$	$C_{36}H_{36}B_2F_8N_{10}Zn$
Formula weight	921.66	841.30	869.60	886.96	847.74
Temperature/K	100(2)	100(2)	100(2)	99.9(3)	100(2)
Crystal system	monoclinic	monoclinic	triclinic	monoclinic	monoclinic
Space group	$P2_1/n$	$P2_1/c$	P1	C2/c	$P2_1/c$
a/Å	13.250(3)	12.280(3)	10.590(2)	15.5514(4)	12.310(3)
b/Å	14.550(3)	17.610(4)	10.880(2)	15.0367(4)	17.650(4)
c/Å	21.590(4)	17.620(4)	17.890(4)	35.2935(8)	17.490(4)
α/°	90	90	104.19(3)	90	90
β/°	102.51(3)	95.18(3)	93.19(3)	103.105(3)	93.67(3)
γ/°	90	90	95.32(3)	90	90
Volume/$Å^3$	4063.4(15)	3794.8(13)	1983.2(7)	8038.1(4)	3792.3(13)
Z	4	4	2	8	4
$\rho_{calc}g/cm^3$	1.507	1.473	1.456	1.466	1.485
μ/mm^{-1}	0.526	0.534	0.571	0.625	0.730
F(000)	1908.0	1724.0	894.0	3640.0	1736.0
Crystal size/mm^3	0.05 × 0.02 × 0.02	0.05 × 0.02 × 0.02	0.1 × 0.1 × 0.05	0.2 × 0.15 × 0.02	0.2 × 0.2 × 0.15
Radiation	MoKα (λ = 0.71073)	MoKα (λ = 0.71073)	MoKα (λ = 0.71073)	MoKα (λ = 0.71073)	MoKα (λ = 0.71073)
2Θ range for data collection/°	3.318 to 57.564	3.276 to 57.542	2.356 to 57.642	5.364 to 58.652	3.282 to 57.59
Index ranges	-17 ≤ h ≤ 17, -19 ≤ k ≤ 19, -29 ≤ l ≤ 25	-16 ≤ h ≤ 16, -23 ≤ k ≤ 22, -23 ≤ l ≤ 23	-13 ≤ h ≤ 13, -14 ≤ k ≤ 14, -24 ≤ l ≤ 23	-20 ≤ h ≤ 21, -20 ≤ k ≤ 19, -47 ≤ l ≤ 48	-16 ≤ h ≤ 16, -23 ≤ k ≤ 23, -23 ≤ l ≤ 23
Reflections collected	75611	70089	37029	36831	70182
Independent reflections	9002 [R_{int} = 0.0236, R_{sigma} = 0.0125]	8365 [R_{int} = 0.0219, R_{sigma} = 0.0121]	15212 [R_{int} = 0.0365, R_{sigma} = 0.0435]	9748 [R_{int} = 0.0785, R_{sigma} = 0.1024]	8300 [R_{int} = 0.0350, R_{sigma} = 0.0175]

Data/restraints/parameters	9002/20/642	8365/0/514	15212/65/1214	9748/104/639	8300/0/515
Goodness-of-fit on F^2	1.026	1.053	1.049	1.072	1.151
Final R indexes [I>=2σ (I)]	R_1 = 0.0531, wR_2 = 0.1595	R_1 = 0.0395, wR_2 = 0.1081	R_1 = 0.0560, wR_2 = 0.1583	R_1 = 0.1124, wR_2 = 0.2313	R_1 = 0.0406, wR_2 = 0.1281
Final R indexes [all data]	R_1 = 0.0570, wR_2 = 0.1640	R_1 = 0.0405, wR_2 = 0.1088	R_1 = 0.0565, wR_2 = 0.1593	R_1 = 0.1840, wR_2 = 0.2691	R_1 = 0.0442, wR_2 = 0.1329
Largest diff. peak/hole / e Å^{-3}	0.56/-0.62	0.74/-0.69	0.98/-0.61	0.63/-0.37	0.67/-0.65
Flack parameter	n/a	n/a	0.027(13)	n/a	n/a

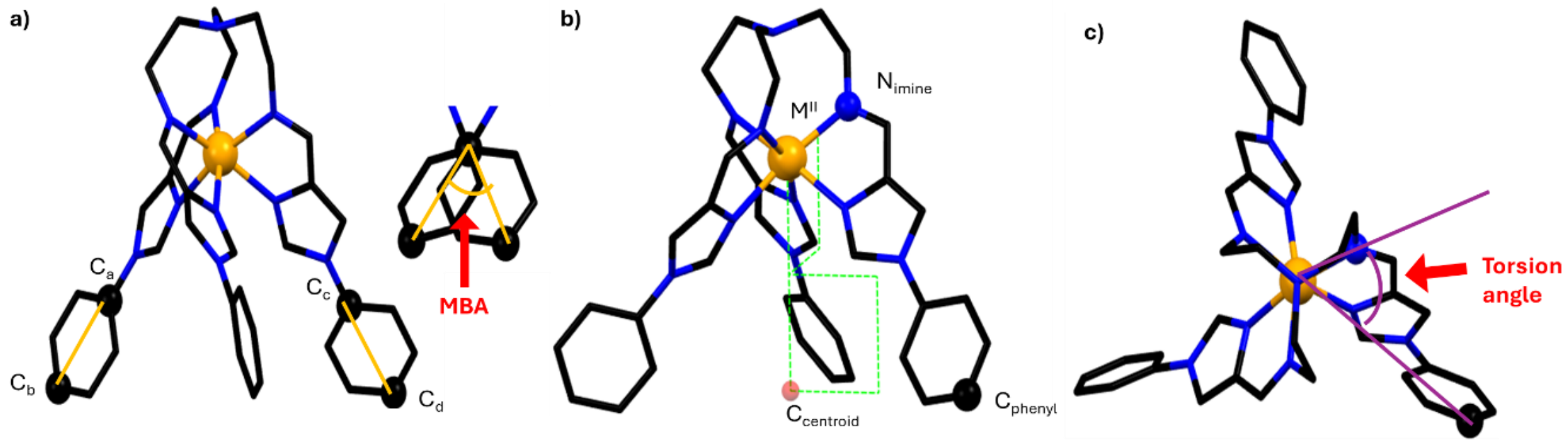

Figure S31: Schematic representation of a) the mutual bonding axis (MBA) and ligand torsion (LT) viewed from the b) side view and c) down the pseudo-C_3 axis

The MBA is calculated by measuring the angle between two vectors generated by the terminal C atom and the opposing C atom in two adjacent phenyl rings in the same complex. The LT is calculated by measuring the torsional angle between C_{phenyl}-$C_{centroid}$-M^{II}-N_{imine} (where $C_{centroid}$ is a point positioned at the midpoint between the three terminal phenyl carbon atoms) and quantifies the degree of twist that the ligand adopts in the metal complex.[5]

References

[1] N. P. Cowieson, D. Aragao, M. Clift, D. J. Ericsson, C. Gee, S. J. Harrop, *et al.*, "MX1: a bending-magnet crystallography beamline serving both chemical and macromolecular crystallography communities at the Australian Synchrotron". *J. Synchrotron Radiat.*, **22**, (2015): 187

[2] G. M. Sheldrick, "Crystal structure refinement with *SHELXL*". *Acta Crystallogrpahica Section C*, **71**, (2015): 3

[3] G. M. Sheldrick, "*SHELXT* – Integrated Space-Group and Crystal-Structure Determination". *Acta Crystallogrpahica Section A Foundations and Advances*, **71**, (2015): 3

[4] G. M. Sheldrick, "SHELX-2014: Programs for Crystal Structure Analysis". *Acta Crystallogrpahica Section A Foundations and Advances*, **71**, (2015): 3

[5] O. V. Dolomanov, L. J. Bourhis, R. J. Gildea, J. A. K. Howard and H. Puschmann, "OLEX2: A complete structure solution, refinement and analysis program". *Journal of Applied Crystallography*, **42**, (2009): 339

[6] H. Min, A. R. Craze, T. Taira, M. J. Wallis, M. M. Bhadbhade, R. Tian, D. J. Fanna, R. Wuhrer, S. Hayami, J. K. Clegg, C. E. Marjo, L. F. Lindoy and F. Li, "Self-Assembly of a Rare High Spin Fe^{II}/Pd^{II} Tetradecanuclear Cubic Cage Constructed via the Metalloligand Approach". *Chemistry*, **4**, (2022): 535
